# Effects of PM_2.5_ and its constituents on hemoglobin during the third trimester in pregnant women

**DOI:** 10.1007/s11356-022-18693-2

**Published:** 2022-01-20

**Authors:** Guilan Xie, Jie Yue, Wenfang Yang, Liren Yang, Mengmeng Xu, Landi Sun, Boxing Zhang, Leqian Guo, Mei Chun Chung

**Affiliations:** 1 Department of Obstetrics and Gynecology, Maternal & Child Health Center, The First Affiliated Hospital of Xi’an Jiaotong University, No. 277, West Yanta Road, Shaanxi Province 710061 Xi’an, People’s Republic of China; 2 School of Public Health, Xi’an Jiaotong University Health Science Center, Xi’an, Shaanxi Province People’s Republic of China; 3 Department of Pediatrics, The First Affiliated Hospital of Xi’an Jiaotong University, Xi’an, Shaanxi Province People’s Republic of China; 4 Division of Nutrition Epidemiology and Data Science, Friedman School of Nutrition Science and Policy, Tufts University, Boston, Massachusetts USA

**Keywords:** PM_2.5_, Constituents of PM_2.5_, Hemoglobin, Anemia, Pregnant women, Third trimester

## Abstract

Anemia has been a public health issue evoking global concern, and the low hemoglobin (Hb) concentration links to adverse pregnancy outcomes. However, the associations of PM_2.5_ and its constituents with Hb and anemia in pregnant women remain unclear. In this retrospective birth cohort study, 7932 pregnant women who delivered in the First Affiliated Hospital of Xi’an Jiaotong University from 2015 to 2018 were included. The Hb during the third trimester in pregnant women was assessed before delivery. PM_2.5_ and its constituents (BC, NH_4_^+^, NO_3_^−^, OM, SO_4_^2−^, and Dust) during pregnancy were retrieved from the V4.CH.03 product constructed by the Atmospheric Composition Analysis Group. Generalized linear regression model was applied to investigate the effects of PM_2.5_ and its constituents on Hb and anemia during the third trimester in pregnant women. The means and standard deviations of PM_2.5_, BC, NH_4_^+^, NO_3_^−^, OM, SO_4_^2−^, and Dust were 69.56 (15.24), 10.02 (2.72), 8.11 (1.77), 14.96 (5.42), 15.36 (4.11), 10.08 (1.20), and 10.98 (1.85) μg/m^3^, respectively. Per IQR increase (μg/m^3^) of PM_2.5_, BC, NO_3_^−^, and OM linked to − 0.75 (− 1.50, − 0.01), − 0.85 (− 1.65, − 0.04), − 0.79 (− 1.56, − 0.03), and − 0.73 (− 1.44, − 0.03) g/L decrease of Hb during the third trimester in multiparous pregnant women, but not for NH_4_^+^, SO_4_^2−^, Dust, and primiparous pregnant women. PM_2.5_ and its constituents had no significant association with anemia, except for Dust (OR: 0.90, 95% CI: 0.82, 0.99, per IQR increase) in primiparous pregnant women. Besides, SO4^2−^ was of lag effects on Hb and anemia in multiparous pregnant women. Moreover, non-linear associations were found among PM_2.5_ and its constituents, Hb, and anemia. Therefore, exposure to PM_2.5_ and some constituents of PM_2.5_ was associated with reduced Hb level during the third trimester in multiparous pregnant women. Related departments and pregnant women should take targeted actions to eliminate the detrimental effects of PM_2.5_ and its constituents on pregnancy outcomes.

**Supplementary Information:**

The online version contains supplementary material available at 10.1007/s11356-022-18693-2.

## Introduction

Nutritional status during pregnancy not only has influence on the health of pregnant women, but also the growth and development of infant. The Developmental Origin of Health and Disease (DOHaD) concept holds that maternal nutritional status links to birth outcomes of infants and the risks of chronic diseases in adulthood via epigenetic regulation, and the health impacts even remain in multiple generations (Gluckman et al. 2016). Hemoglobin (Hb), which plays a role in oxygen transmission and exchange, is one of the proxies that mirror the nutritional status. Anemia is characterized by the decreased concentration of Hb, which could lead to placental dysfunction through influencing the perfusion of placenta and restricting the oxygen exchange between pregnant women and fetus, so as to result in adverse pregnancy outcomes. Previous studies reported that the lower concentration of Hb in pregnant women might increase the risks of gestational hypertension, postpartum hemorrhage, perinatal mortality, preterm birth, low birth weight, and small for gestational age (Ali et al. 2020; Jung et al. 2019; Young et al. 2019).

Anemia has been a public health issue evoking global concern. It was estimated that the prevalence of anemia for pregnant women in the world was 38.2% (WHO 2015). Anemia is attributed to many factors, such as reproductive history, socioeconomic status, and environment (Çelik Kavak & Kavak 2017; Honda et al. 2017; Ullah et al. 2019). The dominant category of anemia in pregnant women is iron deficiency anemia. PM_2.5_ is a complex object containing several constituents with different toxicities. A growing body of epidemiologic studies confirmed that PM_2.5_ had detrimental health impacts on pregnant women and fetuses (Ghassabian et al. 2019; Shang et al. 2019; Xie et al. 2021). PM_2.5_ can be inhaled into alveoli and enter circulatory system by lung ventilation, and then induce inflammatory stress, immune response, and alteration of iron homeostasis, ultimately change Hb level (Badman & Jaffé 1996; Ganz 2019; Seaton et al. 1999; Weiss et al. 2019).

Although there are some researches investigating the association of PM_2.5_ with Hb and anemia, most of them focus on the elderly, adults, and children (Elbarbary et al. 2020; Honda et al. 2017; Mehta et al. 2021; Morales-Ancajima et al. 2019; Wang et al. 2021), and few focus on pregnant women (Liao et al. 2019). Meanwhile, some studies verified the negative associations of PM_2.5_ with Hb and anemia (Elbarbary et al. 2020; Honda et al. 2017; Mehta et al. 2021; Morales-Ancajima et al. 2019), but other studies found the positive associations (Liao et al. 2019; Wang et al. 2021). In addition, there is a knowledge gap in the associations of constituents of PM_2.5_ with Hb and anemia.

To address the gap, we were calculated to figure out the effects of PM_2.5_ and its constituents on Hb and anemia during the third trimester in pregnant women. It would effectively assist targeted regulatory control and individual protection, so as to minimize the detrimental effects of PM_2.5_ and its constituents on pregnant outcomes.

## Methods

### Study population

In this retrospective birth cohort study, Chinese pregnant women who delivered in the First Affiliated Hospital of Xi’an Jiaotong University from 2015 to 2018 and lived in Xi’an, China, during pregnancy were included. All information of pregnant women was retrieved from medical record. The excluded criteria contained (1) aged younger than 20 years or older than 45 years old, because the legal minimum marriage age for women in China was 20 years old (Hare-Mustin 1982) and it was very hard to conceive naturally for women who were older than 45 years old owing to the declined fertility ability (Forman et al. 2011); (2) with kidney diseases; (3) without results of blood examination about the concentration of Hb before delivery; and (4) gestational age was less than 28 weeks when doing the blood examination. The flowchart of participants’ selection was shown in Fig. S1. Basic characteristics of the included and excluded pregnant women were of no significant differences, except for gestational diabetes (Table S1). Locations of residential address of pregnant women and hospital in the birth cohort were visualized in Fig. S2. This study was approved by the Medical Ethics Committee of the First Affiliated Hospital of Xi’an Jiaotong University (No. XJTU1AF2020LSK-261).

Gestational age was calculated by subtracting last menstrual period from the date of blood examination before delivery. Sociodemographic information, including age (years old), gestational age (weeks), gestational weight gain (GWG) (kilogram), ethnicity (Han and minorities), educational level (≤ 9, 10–12, and > 12 years old), occupation (farmer, worker, others, and none), gestational hypertensive disorders (yes or no), and gestational diabetes (yes or no), was collected by qualified obstetricians, midwives, and nurses. GDP and population density were extracted from the raster images of Resource and Environment Science and Data Center (https://www.resdc.cn/) and WorldPop (https://www.worldpop.org/) with 1000 m buffer based on the resident addresses of pregnant women through QGIS 3.14 (https://www.qgis.org/en/site/). GDP and population density were the region-level data for the region where pregnant women lived during pregnancy, and they were divided into the low and high groups according to medians. Generally, Xi’an conducted heat warning from November to March. Considering burning coal for heat warning was the main source of PM_2.5_ and its constituent, and Hb level varied with seasons, season of blood examination was categorized as warm season (from April to October) and cold season (from November to March). Directed acyclic graph for PM_2.5_ and its constituents, Hb and anemia, and covariables was shown in Fig. S3.

### Assessment of Hb and anemia

Venous blood of 2 mL was collected into the tube added with ethylenediaminetetraacetic acid when pregnant women were admitted into the hospital before delivery. The blood samples were mixed upside down and sent to the standard clinical laboratory within 2 h to assess the concentration of Hb. And the blood examination was done by the Sysmex XN-3000 automated hematology analyzer (Sysmex Corporation, Kobe, Japan). The diagnostic standard of anemia for pregnant women was that concentration of Hb was lower than 110 g/L (WHO 2011).

### *Assessment of PM*_*2.5*_* and its constituents*

The month-level data of PM_2.5_ and its constituents from 2014 to 2018 were gained from the monthly gridded dataset of V4.CH.03 (*R*^2^ = 0.71, RMSE = 9.3 μg/m^3^) with a resolution of 0.01° × 0.01° and constructed by the Atmospheric Composition Analysis Group (https://sites.wustl.edu/acag/). The concentrations of PM_2.5_ and its constituents were estimated based on the satellite remote sensing data and calibrated with the ground monitoring data (Hammer et al. 2020; van Donkelaar et al. 2021, 2019). In this study, the constituents of PM_2.5_ included black carbon (BC), ammonium (NH_4_^+^), nitrate (NO_3_^−^), organic matter (OM), sulfate (SO_4_^2−^), and mineral dust (Dust). PM_2.5_ and its constituents for each pregnant woman were extracted based on the longitude and latitude corresponding to the resident address of pregnant woman with 1000 m buffer through QGIS 3.14. And the mean concentrations of PM_2.5_ and its constituents during the period between last menstrual period and the date of blood examination before delivery for each pregnant woman were calculated through time-weighted method (Lin et al. 2020; Zhao et al. 2020).

### Statistical analysis

Mean and standard deviation were conducted to display the distributions of normal-distributed continuous variables. Median and interquartile range (IQR) were used to describe the skewed-distributed continuous variables. Frequency and proportion were used to describe the category variables. Student’s *t*-test and chi-square analysis were adopted to identify differences between the primiparous and multiparous women. Spearman correlation was deployed to find the correlations of PM_2.5_ and its constituents. Generalized linear regression model was applied to investigate the effects of PM_2.5_ and its constituents on Hb and anemia during the third trimester in pregnant women. PM_2.5_ and its constituents were included as independent variables in the one pollution model, respectively. Besides, as red blood cells updated every 120 days, the lag effects of PM_2.5_ and its constituents on Hb and anemia were investigated by the moving average concentrations of PM_2.5_ and its constituents on the day of blood examination and 1, 2, 3, 4 months prior to blood examination (from lag01 to lag04 months). Furthermore, restricted cubic spline was applied to explore the non-linear associations of PM_2.5_ and its constituents with Hb and anemia.

### Sensitive analysis

First, the associations were further stratified by sociodemographic variables and the modifications of sociodemographic variables were assessed with the multiplicative terms. In the stratified analysis, age, gestational age, and GWG were divided into subgroups. Second, the associations of ground monitor based PM_2.5_ with Hb and anemia were further analyzed. The PM_2.5_ data of 13 ground monitoring stations in Xi’an was collected from the Ministry of Ecology and Environment of the People’s Republic of China (http://www.mee.gov.cn/). And the ground monitor based PM_2.5_ exposure was assigned according to the residential addresses of pregnant women by inverse distance weighting method.

Package of “rms” for R 3.6.1 (https://www.r-project.org/) was applied and *p* < 0.05 was considered statistically significant.

## Results

### Baseline characteristics

A total of 7932 pregnant women were included in this study, among which there were 5323 primiparous pregnant women and 2609 multiparous pregnant women. Baseline characteristics of the birth cohort were depicted in Table 1. Mean age of primiparous pregnant women was 28.78 ± 3.25 years old, and 32.77 ± 3.75 years old for multiparous pregnant women. Mean gestational age for primiparous and multiparous pregnant women were 39.00 ± 1.73 weeks and 38.35 ± 1.89 weeks, respectively. Mean GWG for primiparous and multiparous pregnant women were 15.70 ± 4.53 kg and 14.46 ± 4.48 kg, respectively. Mean Hb concentration for primiparous and multiparous pregnant women were 119.53 ± 13.12 g/L and 116.16 ± 13.90 g/L. The prevalence of anemia was 22.17% and 29.24% for primiparous and multiparous pregnant women. The differences of age, gestational age, GWG, educational level, occupation, gestational diabetes, concentration of Hb, and anemia between primiparous and multiparous pregnant women were statistically significant (*p* < 0.05).

**Table 1:** Baseline characteristics of the birth cohort

Variables	Total (*n* = 7932)	Primipara (*n* = 5323)	Multipara (*n* = 2609)	*t*/χ^2^	*p*
Age, years old	30.10 ± 3.90	28.78 ± 3.25	32.77 ± 3.75	− 46.45^a^	< 0.001
Gestational age, weeks	38.79 ± 1.81	39.00 ± 1.73	38.35 ± 1.89	14.80^a^	< 0.001
GWG	15.29 ± 4.55	15.70 ± 4.53	14.46 ± 4.48	11.41^a^	< 0.001
Ethnicity				2.91^b^	0.09
Han	7874 (99.27)	5278 (99.15)	2596 (99.50)		
Minorities	58 (0.73)	45 (0.85)	13 (0.50)		
Educational level, years^c^				231.90^b^	< 0.001
≤ 9	572 (7.23)	255 (4.80)	317 (12.19)		
10–12	633 (8.00)	329 (6.20)	304 (11.69)		
> 12	6705 (84.77)	4726 (89.00)	1979 (76.12)		
Occupation				71.43^b^	< 0.001
Farmer	231 (2.91)	114 (2.14)	117 (4.48)		
Worker	1838 (23.17)	1335 (25.08)	503 (19.28)		
Others	4937 (62.24)	3304 (62.07)	1633 (62.59)		
None	926 (11.68)	570 (10.71)	356 (13.65)		
Gestational hypertensive disorders				2.10^b^	0.15
Yes	479 (6.04)	307 (5.77)	172 (6.59)		
No	7453 (93.96)	5016 (94.23)	2437 (93.41)		
Gestational diabetes				16.10^b^	< 0.001
Yes	543 (6.85)	322 (6.05)	221 (8.47)		
No	7389 (93.15)	5001 (93.95)	2388 (91.53)		
GDP					
Low	3965 (49.99)	2665 (50.07)	1300 (49.83)	0.04^b^	0.84
High	3967 (50.01)	2658 (49.93)	1309 (50.17)		
Population density					
Low	3966 (50.00)	2675 (50.25)	1291 (49.48)	0.42^b^	0.52
High	3966 (50.00)	2648 (49.75)	1318 (50.52)		
Season of blood examination					
Warm season	4657 (58.71)	3128 (58.76)	1529 (58.60)	0.02^b^	0.89
Cold season	3275 (41.29)	2195 (41.24)	1080 (41.40)		
Hb, g/L	118.42 ± 13.47	119.53 ± 13.12	116.16 ± 13.90	10.36^a^	< 0.001
Anemia				47.41^b^	< 0.001
Yes	1943 (24.50)	1180 (22.17)	763 (29.24)		
No	5989 (75.50)	4143 (77.83)	1846 (70.76)		

Note: ^a^ indicated Student’s *t*-test, ^b^ indicated chi-square analysis, ^c^ *n* = 7910 for total pregnant women, *n* = 5310 for primipara, and *n* = 2600 for multipara in educational level

### *Distributions of PM*_*2.5*_* and its constituents*

The distributions of PM_2.5_ and its constituents were shown in Table 2. For total pregnant women, the means and standard deviations of PM_2.5_, BC, NH_4_^+^, NO_3_^−^, OM, SO_4_^2−^, and Dust were 69.56 (15.24), 10.02 (2.72), 8.11 (1.77), 14.96 (5.42), 15.36 (4.11), 10.08 (1.20), and 10.98 (1.85) μg/m^3^, respectively. For primiparous pregnant women, the means and standard deviations of PM_2.5_, BC, NH_4_^+^, NO_3_^−^, OM, SO_4_^2−^, and Dust were 68.72 (14.60), 9.88 (2.62), 8.01 (1.70), 14.67 (5.20), 15.19 (3.93), 10.05 (1.21), and 10.88 (1.82) μg/m^3^, respectively. For multiparous pregnant women, the means and standard deviations of PM_2.5_, BC, NH_4_^+^, NO_3_^−^, OM, SO_4_^2−^, and Dust were 71.26 (16.34), 10.31 (2.90), 8.31 (1.90), 15.57 (5.80), 15.70 (4.44), 10.16 (1.17), and 11.17 (1.91) μg/m^3^, respectively. PM_2.5_ and its constituents were positively correlated both in primiparous and multiparous pregnant women, but SO_4_^2−^ and Dust were negatively correlated (Table S2).

**Table 2:** Distributions of PM_2.5_ and its constituents (μg/m^3^) of the birth cohort

Variables	Mean (SD)	IQR	Min	25th	50th	75th	Max
Total							
PM_2.5_	69.56 (15.24)	19.37	8.32	58.72	67.00	78.09	125.75
BC	10.02 (2.72)	3.88	1.06	7.90	9.77	11.78	19.65
NH_4_^+^	8.11 (1.77)	2.04	1.15	6.87	7.89	8.91	14.76
NO_3_^−^	14.96 (5.42)	7.46	1.07	10.62	14.28	18.08	35.26
OM	15.36 (4.11)	5.00	1.74	12.37	15.16	17.37	29.85
SO_4_^2−^	10.08 (1.20)	1.59	2.12	9.24	10.00	10.83	14.00
Dust	10.98 (1.85)	2.45	1.04	9.63	10.80	12.08	17.26
Primipara							
PM_2.5_	68.72 (14.60)	18.05	13.15	58.62	65.93	76.67	125.47
BC	9.88 (2.62)	3.69	1.61	7.87	9.54	11.56	19.30
NH_4_^+^	8.01 (1.70)	1.91	1.52	6.86	7.83	8.77	14.35
NO_3_^−^	14.67 (5.20)	7.05	2.10	10.53	13.92	17.58	34.82
OM	15.19 (3.93)	4.79	2.56	12.38	14.97	17.17	29.51
SO_4_^2−^	10.05 (1.21)	1.58	2.43	9.21	9.95	10.79	14.00
Dust	10.88 (1.82)	2.39	2.50	9.58	10.70	11.97	16.64
Multipara							
PM_2.5_	71.26 (16.34)	21.85	8.32	58.99	69.18	80.84	125.75
BC	10.31 (2.90)	4.17	1.06	8.04	10.29	12.21	19.65
NH_4_^+^	8.31 (1.90)	2.30	1.15	6.93	8.11	9.23	14.76
NO_3_^−^	15.57 (5.80)	8.01	1.07	10.88	15.14	18.89	35.26
OM	15.70 (4.44)	5.50	1.74	12.36	15.66	17.86	29.85
SO_4_^2−^	10.16 (1.17)	1.62	2.12	9.29	10.11	10.91	13.54
Dust	11.17 (1.91)	2.51	1.04	9.79	10.99	12.30	17.26

### *Linear associations of PM*_*2.5*_* and its constituents with Hb and anemia*

**Fig. 1:**
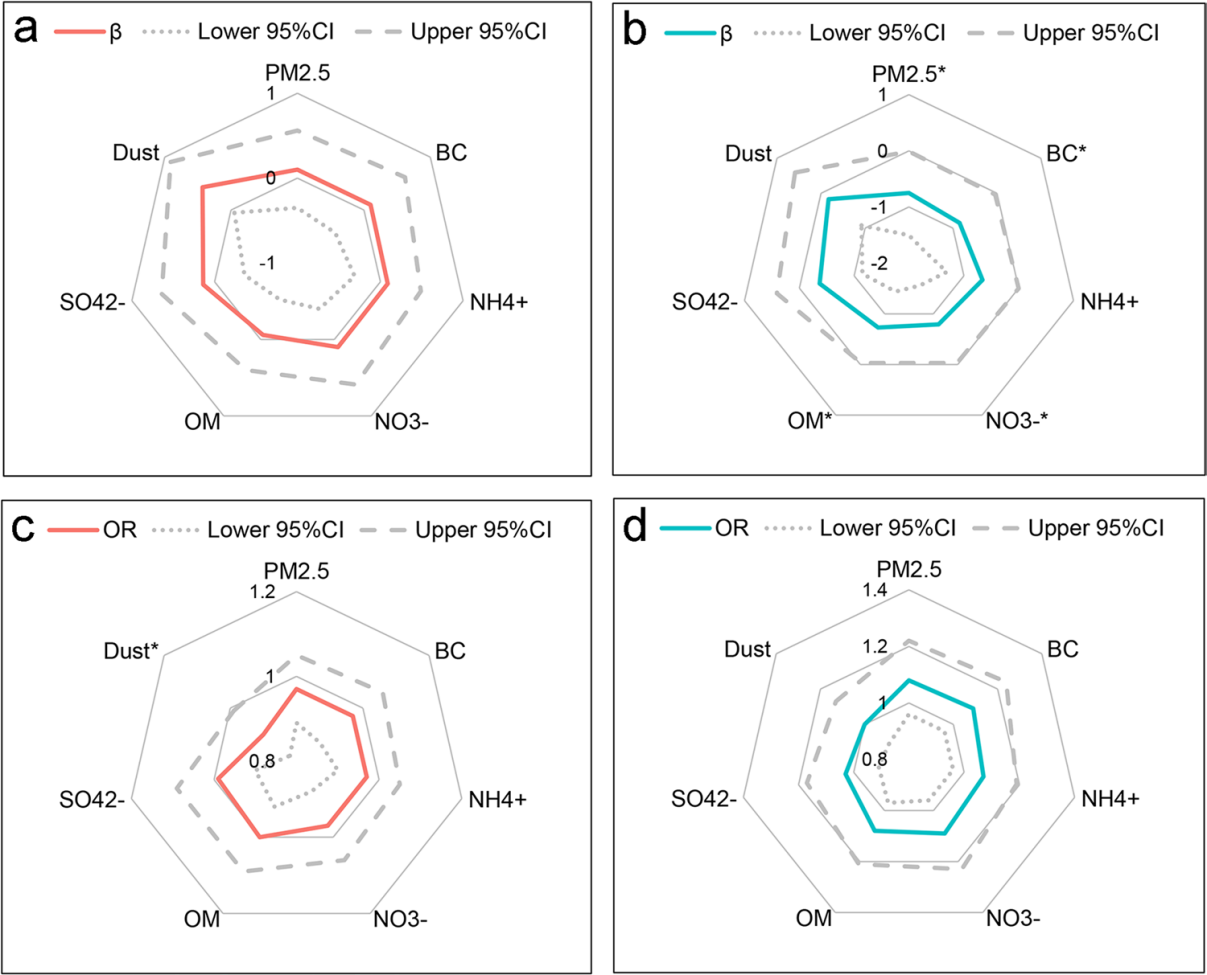
Linear associations of per IQR increase (μg/m^3^) of PM_2.5_ and its constituents with Hb and anemia. The linear associations of PM_2.5_ and its constituents with Hb in primipara (**a**) and multipara (**b**). The linear associations of PM_2.5_ and its constituents with anemia in primipara (**c**) and multipara (**d**). * indicated *p* < 0.05. Adjusted for age, gestational age, GWG, ethnicity, educational level, occupation, gestational hypertensive disorders, gestational diabetes, GDP, population density, and season of blood examination

The linear associations of per IQR increase (μg/m^3^) of PM_2.5_ and its constituents with Hb and anemia were provided in Fig. 1. PM_2.5_ and its constituents and Hb had no significant association in primiparous pregnant women. For multiparous pregnant women, BC was of the highest effect on Hb (*β*: − 0.85, 95% CI: − 1.65, − 0.04, g/L, per IQR increase) after adjusting for age, gestational age, GWG, ethnicity, educational level, occupation, gestational hypertensive disorders, gestational diabetes, GDP, population density, and season of blood examination, which was in line with the general knowledge that BC was the most harmful component of PM_2.5_. Following by BC, per IQR increase (μg/m^3^) of NO_3_^−^, PM_2.5_, and OM linked to − 0.79 (− 1.56, − 0.03), − 0.75 (− 1.50, − 0.01), and − 0.73 (− 1.44, − 0.03) g/L decrease of Hb during the third trimester, but not for NH_4_^+^, SO_4_^2−^, and Dust. Likely, PM_2.5_ and its constituents and anemia had no significant association in primiparous pregnant women, except for Dust (OR: 0.90, 95% CI: 0.82, 0.99, per IQR increase). However, there was no significant association of PM_2.5_ and its constituents with anemia being found in multiparous pregnant women.

### *Lag effects of PM*_*2.5*_* and its constituents on Hb and anemia*

The lag effects of PM_2.5_ and its constituents (μg/m^3^) on Hb and anemia were displayed in Table 3. The lag effects of PM_2.5_ and its constituents on Hb and anemia were not observed in primiparous pregnant women. Per IQR increase of SO_4_^2−^ at lag01 month decreased the Hb concentration (*β*: − 0.38, 95% CI: − 0.75, − 0.01, g/L) and per IQR increase of SO_4_^2−^ at lag01, lag02, and lag03 months increased the risk of anemia (OR: 1.09–1.10) in multiparous pregnant women, while lag effects of PM_2.5_, BC, NH_4_^+^, NO_3_^−^, OM, and Dust on Hb and anemia were not identified in other lag models for multiparous pregnant women.

**Table 3:** Lag effects of PM_2.5_ and its constituents (μg/m^3^) on Hb and anemia

Variables	Primipara [*β*/OR (95% CI)]				Multipara [*β*/OR (95% CI)]			
	Lag01	Lag02	Lag03	Lag04	Lag01	Lag02	Lag03	Lag04
Hb								
PM_2.5_	− 0.02 (− 0.27, 0.23)	− 0.03 (− 0.28, 0.23)	− 0.05 (− 0.29, 0.19)	− 0.08 (− 0.31, 0.16)	− 0.12 (− 0.56, 0.32)	− 0.25 (− 0.68,0.19)	− 0.33 (− 0.74, 0.09)	− 0.34 (− 0.74, 0.05)
BC	0.01 (− 0.28, 0.29)	0.03 (− 0.25, 0.32)	0.01 (− 0.26, 0.28)	− 0.04 (− 0.30, 0.22)	− 0.06 (− 0.54, 0.41)	− 0.22 (− 0.68, 0.25)	− 0.29 (− 0.73, 0.14)	− 0.30 (− 0.72, 0.12)
NH_4_^+^	− 0.03 (− 0.26, 0.19)	− 0.01 (− 0.24, 0.23)	− 0.02 (− 0.25, 0.22)	− 0.05 (− 0.28, 0.19)	− 0.16 (− 0.55, 0.23)	− 0.28 (− 0.69, 0.13)	− 0.36 (− 0.76, 0.05)	− 0.35 (− 0.74, 0.05)
NO_3_^−^	− 0.01 (− 0.29, 0.28)	0.02 (− 0.27, 0.30)	− 0.01 (− 0.28, 0.26)	− 0.04 (− 0.30, 0.22)	− 0.07 (− 0.53, 0.40)	− 0.24 (− 0.71, 0.22)	− 0.33 (− 0.76, 0.11)	− 0.32 (− 0.73, 0.10)
OM	− 0.06 (− 0.28, 0.16)	− 0.02 (− 0.24, 0.20)	− 0.04 (− 0.25, 0.17)	− 0.08 (− 0.30, 0.13)	− 0.11 (− 0.47, 0.26)	− 0.21 (− 0.57, 0.15)	− 0.27 (− 0.62, 0.08)	− 0.28 (− 0.62, 0.07)
SO_4_^2−^	− 0.11 (− 0.35, 0.13)	− 0.07 (− 0.34, 0.19)	− 0.05 (− 0.34, 0.24)	− 0.07 (− 0.39, 0.25)	− 0.38 (− 0.75, − 0.01)	− 0.39 (− 0.81, 0.03)	− 0.38 (− 0.84, 0.08)	− 0.36 (− 0.87, 0.16)
Dust	0.03 (− 0.09, 0.16)	− 0.06 (− 0.20, 0.09)	− 0.12 (− 0.30, 0.06)	− 0.15 (− 0.38, 0.08)	0.02 (− 0.17, 0.21)	0.02 (− 0.20, 0.25)	− 0.05 (− 0.32, 0.23)	− 0.25 (− 0.60, 0.09)
Anemia								
PM_2.5_	0.97 (0.93, 1.02)	0.98 (0.93, 1.03)	1.00 (0.95, 1.04)	1.01 (0.97, 1.05)	1.06 (0.99, 1.14)	1.06 (0.98, 1.13)	1.05 (0.98, 1.12)	1.05 (0.98, 1.11)
BC	0.97 (0.92, 1.02)	0.97 (0.92, 1.03)	0.99 (0.94, 1.04)	1.01 (0.96, 1.06)	1.03 (0.96, 1.11)	1.03 (0.96, 1.12)	1.04 (0.96, 1.11)	1.04 (0.97, 1.11)
NH_4_^+^	0.98 (0.93, 1.02)	0.97 (0.93, 1.02)	0.99 (0.95, 1.04)	1.01 (0.96, 1.05)	1.06 (1.00, 1.13)	1.06 (0.99, 1.13)	1.06 (0.99, 1.13)	1.05 (0.98, 1.12)
NO_3_^−^	0.97 (0.91, 1.02)	0.97 (0.92, 1.03)	0.99 (0.94, 1.05)	1.01 (0.96, 1.06)	1.05 (0.97, 1.13)	1.05 (0.97, 1.13)	1.04 (0.97, 1.12)	1.04 (0.97, 1.11)
OM	0.99 (0.95, 1.03)	0.99 (0.95, 1.03)	1.00 (0.96, 1.05)	1.02 (0.98, 1.06)	1.03 (0.97, 1.10)	1.03 (0.97, 1.09)	1.03 (0.98, 1.09)	1.03 (0.98, 1.09)
SO_4_^2−^	1.00 (0.95, 1.04)	0.98 (0.93, 1.03)	0.98 (0.93, 1.04)	0.99 (0.94, 1.05)	1.09 (1.03, 1.16)	1.10 (1.03, 1.17)	1.10 (1.02, 1.18)	1.09 (1.00, 1.18)
Dust	0.99 (0.97, 1.01)	1.00 (0.97, 1.03)	1.01 (0.97, 1.04)	1.00 (0.96, 1.05)	1.01 (0.98, 1.05)	1.02 (0.98, 1.05)	1.02 (0.98, 1.07)	1.04 (0.98, 1.10)

Note: Adjusted for age, gestational age, GWG, ethnicity, educational level, occupation, gestational hypertensive disorders, gestational diabetes, GDP, population density, and season of blood examination

### *Non-linear associations of PM*_*2.5*_* and its constituents with Hb and anemia*

**Fig. 2:**
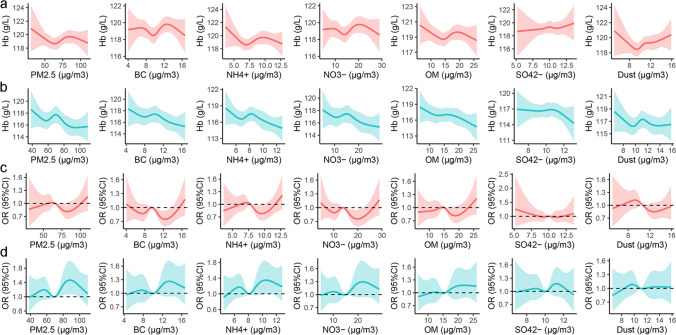
Non-linear associations of PM_2.5_ and its constituents with Hb and anemia. The non-linear associations of PM_2.5_ and its constituents with Hb in primipara (**a**) and multipara (**b**). The non-linear associations of PM_2.5_ and its constituents with anemia in primipara (**c**) and multipara (**d**). Adjusted for age, gestational age, GWG, ethnicity, educational level, occupation, gestational hypertensive disorders, gestational diabetes, GDP, population density, and season of blood examination

The non-linear associations of PM_2.5_ and its constituents with Hb and anemia were showed in Fig. 2. Summarily, there were declined trends of Hb with the increase of PM_2.5_ and its constituents both in primiparous and multiparous pregnant women and the trends slightly fluctuated at the moderate concentrations of PM_2.5_ and its constituents. The associations of PM_2.5_ and its constituents with anemia were “W” and “M” shapes in primiparous and multiparous pregnant women.

### Sensitive analysis

Gestational age, GWG, educational level, ethnicity, occupation, gestational hypertensive disorders, gestational diabetes, population density, and season of blood examination were of modification effects on the associations of PM_2.5_ and its constituents with Hb (Table S3-9) and anemia (Table S10-16). Additionally, our findings were robust in the further analyses with ground monitor based PM_2.5_ (Table S17-21, Fig. S4).

## Discussion

We comprehensively explored the effects of PM_2.5_ and its constituents on Hb and anemia during the third trimester in pregnant women. Increased PM_2.5_, BC, NO_3_^−^, and OM were tied to the declined Hb level in multiparous pregnant women, but not for NH_4_^+^, SO_4_^2−^, Dust, and primiparous pregnant women. The effects of PM_2.5_ and its constituents on Hb were slight, that was why the significant associations tended to be found in Hb, but not in anemia. Besides, SO_4_^2−^ was of lag effect on Hb and anemia in multiparous pregnant women. Moreover, non-linear associations were found among PM_2.5_ and its constituents, Hb, and anemia.

PM_2.5_ is a sophisticated mixture encompassing many constituents with different sources and toxicities. PM_2.5_ mainly comes from fossil fuel combustion, such as vehicle emission, power station, and factories. Each source might generate several constituents of PM_2.5_, and each constituent of PM_2.5_ might also come from several resources. The toxicities of constituents of PM_2.5_ not only are ascribed to their own effects, but also the interactive effects among them (Kelly & Fussell 2012). In this study, exposure to PM_2.5_ reduced the Hb level (*β*: − 0.75, 95% CI: − 1.50, − 0.01, g/L, per IQR increase of PM_2.5_ in multiparous pregnant women), which was in lined with the previous researches. A representative cohort study in America observed that 1-year moving average PM_2.5_ linked to − 0.81 g/dL decrease of Hb level in older adults, and the dose–response relationship was strong (Honda et al. 2017). Another longitude study organized by WHO indicated that 3-year moving average PM_2.5_ was negatively correlated with the concentration of Hb in older Chinese adults (*β*: − 0.52, 95% CI: − 0.71, − 0.33, g/L, per IQR increase of PM_2.5_) (Elbarbary et al. 2020). Likely, one study for children found that increased exposure to PM_2.5_ was tied to a slight decrease of Hb concentration (*β*: − 0.14, 95% CI: − 0.16, − 0.12, g/L, per 10 μg/m^3^ increase of PM_2.5_) (Mehta et al. 2021). Meanwhile, there were discrepancies in the association between PM_2.5_ and Hb. Conversely, a study revealed that Hb concentration increased with the rise of daily average concentration of PM_2.5_ (*β*: 9.923%, 95% CI: 8.741%, 11.264%, g/L, per 10 μg/m^3^ increase of PM_2.5_) (Wang et al. 2021) and one study for pregnant women illuminated that prenatal exposure to PM_2.5_ linked to higher concentration of Hb (*β*: 0.929, 95% CI: 0.068, 1.789, g/L, per IQR increase of PM_2.5_) (Liao et al. 2019). These discrepancies might be due to the heterogeneities of population, regions, exposure periods, and constituents of PM_2.5_. The chronic exposure to PM_2.5_ might influence the Hb level by interfering the synthesis of Hb, and the acute exposure to PM_2.5_ might accelerate the apoptosis of RBC by upregulating the gene expression of cytokines (Gao et al. 2021).

A systematic review and meta-analysis concluded that BC and organic carbon (OC) were the important harmful constituents of PM_2.5_, and the other constituents of PM_2.5_ also had adverse health effects (Yang et al. 2019). Although there were uncertainties, Kelly and Fussell (2015) concluded that NH_4_^+^, SO_4_^2−^, and NO_3_^−^ had small threats to health. A study for normal people indicated that Hb was negatively correlated with NH_4_^+^, NO_3_^−^, and SO_4_^2−^ in fine particulate matter (Wang et al. 2021). An animal study elucidated that short-term exposure to diesel exhaust particles caused reduction of Hb concentration in rats (Nemmar and Inuwa, 2008). A study recruited 38 healthy subjects exposure to secondary organic aerosol mixed with water-soluble objects led to 8–10% declines in RBC proteasome activity (Kipen et al. 2011). We also found that BC, NO_3_^−^, and OM were tied to lower Hb level in this study. In addition, a handful of studies implied that prenatal exposure to BC, OC, SO_4_^2−^, NH_4_^+^, NO_3_^−^, and Dust were the risk factors for adverse pregnancy outcomes (Cai et al. 2020; Ebisu et al. 2018; Fong et al. 2019).

Pregnancy, breastfeeding, and monthly menstrual cycles contribute to the loss of iron in women, which lead to that multiparous women tend to have higher risk of anemia in pregnancy. A study including 426 pregnant women in Turkey discovered that the nulliparous pregnant women had higher proportion of those with normal hemoglobin values than the multiparous pregnant women (Çelik Kavak & Kavak 2017). Al-Farsi et al. (2011) also observed that the risk ratios of anemia in pregnancy increased as the level of parity increased. During pregnancy, the plasma volume of women increases and induces hemodilution especially in the 32–34 weeks of pregnancy (Cunningham et al. 2014). The prevalence of anemia in pregnancy is affected by sociodemographic factors. The previous studies indicated that pregnant women who were ethnic minorities, with lower educational level and income had higher risk of anemia (Gebre & Mulugeta 2015; Taner et al. 2015; Wu et al. 2020). In general, regions with high population density are coupled with more severe air pollution because of the numerous vehicles, power generation, and road dust.

PM_2.5_ can induce local pulmonary inflammation, and consequently evoke systemic oxidative stress and inflammation, which have been established as the main mechanism of the adverse health effects of PM_2.5_ (Feng et al. 2016; Hiraiwa & van Eeden 2013). PM_2.5_ can induce nephrotoxicity and kidney damage, and then lower the secretion of erythropoietin (EPO) (Hsu et al. 2019; Shih et al. 2018; Zeisberg & Kalluri 2015). The downregulated EPO undermines the proliferation and differentiation of RBC. In addition, PM_2.5_ might exacerbate the erythropoietin resistance and attenuate the hematopoietic function in bone marrow (Bárány 2001; Ganz 2019). Most importantly, PM_2.5_ can cause immune activation and inflammatory stress, and enable the inflammatory factors and cytokines rise, such as C-reactive protein and IL-6, which have impacts on the hepcidin-ferroportin axis (D'Angelo 2013; Nemeth & Ganz 2014). The IL-6 can upregulate hepcidin by the STAT3 signaling in hepatocytes, and the hepcidin bind with ferroportin on cell membrane, so as to induce the internalization and degradation of ferroportin, which hinders the absorption of dietary iron in duodenum, blocks the recycling of the iron from the aging RBC via iron sequestration in macrophages, and the iron might retain in the reticuloendothelial cells (Langer and Ginzburg, 2017; Weiss et al. 2019). The altered iron homeostasis finally interferes the synthesis of Hb.

Our study had some strengths. First, we not only verified the associations of PM_2.5_ with Hb and anemia in pregnant women, but also the associations of constituents of PM_2.5_ with Hb and anemia, which added to the evidences of adverse health impacts of PM_2.5_ and its constituents, and helped to make targeted and effective policies. Second, the lag effects of PM_2.5_ and its constituents on Hb and anemia in pregnant women were investigated, which could aid pregnant women to figure out the sensitive window, timely use air purify equipment, and wear mask. Third, in addition to the linear associations of PM_2.5_ and its constituents with Hb and anemia, the non-linear associations were also explored, which could guide further studies to verify the threshold effects and drive the abatement of air pollution.

Besides, there were some limitations in this study. First, although ambient PM_2.5_ and its constituents are widespread and extensive, the indoor air pollution is also of important health impact on pregnant women. Ali et al. (2021) reviewed that the concentrations of pollutants were higher in indoor environment and they could increase the health risks in women and children through oxidative, DNA methylation, and gene activation. Therefore, failing to obtain the status of indoor pollution, such as household energy, cooking habits, and devices, was a limitation in this retrospective cohort study. Second, family income is related to the nutrition status and antenatal care access of pregnant women. Those with lower family income tend to have higher prevalence of anemia (Kare & Gujo 2021), which might be ascribed to the imbalance dietary patterns and less antenatal visits missing timely intervention. Thus, in spite of the regional-level GDP and population density were adjusted in this study, which could mirror the socioeconomic status of the pregnant women to some extent, the effect of family income was still warranted to be considered. Third, dietary pattern and nutrient supplement could closely influence on the nutritional status of pregnant women. Previous studies (Abriha et al. 2014; Mayasari et al. 2021) indicated that improved meat consumption could help to mitigate the prevalence of anemia in pregnant women and intakes of carbohydrates and vegetables were correlated with gestational iron status by adjusting hepcidin levels. Hence, although gestational weight gain was included to embody the nutritional status of pregnant women, the dietary pattern and nutrient supplement were still critical confounders of the associations of PM_2.5_ and its constituents with Hb and anemia.

## Conclusions

Exposure to PM_2.5_ and some constituents of PM_2.5_ was associated with reduced Hb level during the third trimester in multiparous pregnant women. Related departments should restrict the pirate cars, set standards for the used fossil fuel, and increase the greenness in the urban regions to alleviate the generation of PM_2.5_ and its constituents. Pregnant women should use air purify and put masks when the air pollution is severe to mitigate the inhalation of PM_2.5_ and its constituents, ultimately eliminate their detrimental effects.

## Supplementary Information

Below is the link to the electronic supplementary material.

Supplementary file1 (DOCX 752 KB)

## Data Availability

The data was available from the corresponding author on reasonable request.
