# Supplementary material for: Effects of PM2.5 and its constituents on hemoglobin during the third trimester in pregnant women
Source: Environ Sci Pollut Res Int. 2022 Jan 20;29(23):35193–203. doi: 10.1007/s11356-022-18693-2 (PMC9076737; doi:10.1007/s11356-022-18693-2)
Supplement: Supplementary file 1 — Supplementary file1 (DOCX 752 KB) [file 11356_2022_18693_MOESM1_ESM.docx]

**FEffects of PM_2.5_ and its constituents on hemoglobin during the third trimester in pregnant women**

Guilan Xie ^1,2^ · Jie Yue ^3^ · Wenfang Yang ^1^ · Liren Yang ^1,2^ · Mengmeng Xu ^1^ · Landi Sun ^1,2^ · Boxing Zhang ^1,2^ · Leqian Guo ^1^ · Mei Chun Chung ^4^

**^1^** Department of Obstetrics and Gynecology, Maternal & Child Health Center, The First Affiliated Hospital of Xi’an Jiaotong University, Xi’an, Shaanxi Province, People’s Republic of China

^2^ School of Public Health, Xi’an Jiaotong University Health Science Center, Xi’an, Shaanxi Province, People’s Republic of China

^3^ Department of Pediatrics, The First Affiliated Hospital of Xi’an Jiaotong University, Xi’an, Shaanxi Province, People’s Republic of China

^4^ Division of Nutrition Epidemiology and Data Science, Friedman School of Nutrition Science and Policy, Tufts University, Massachusetts Boston, USA

Guilan Xie and Jie Yue contributed equally to this work.


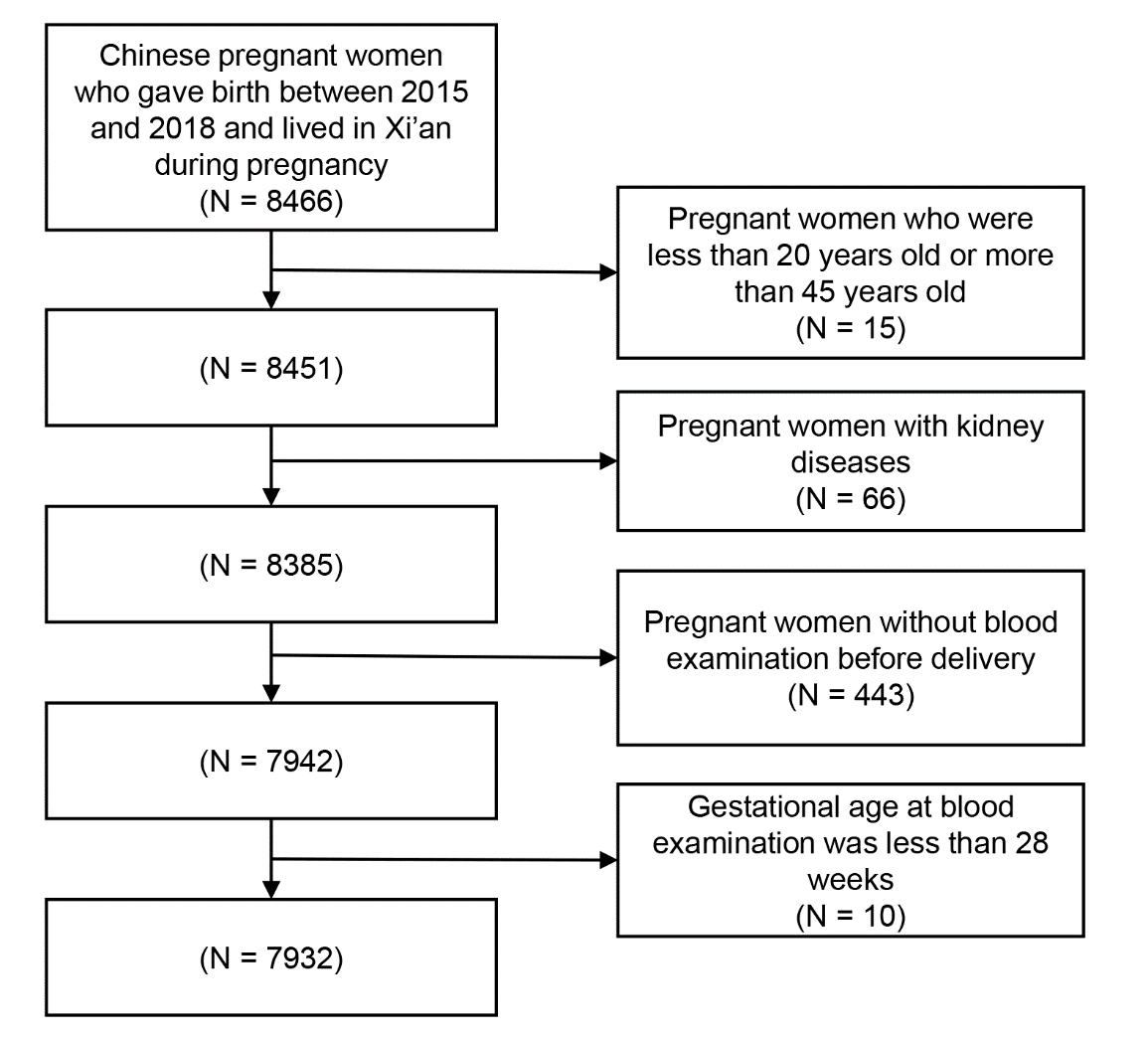


**Fig. S1** Flowchart of participants’ selection

**Table S1** Comparison of basic characteristics of the included and excluded pregnant women

| Variables | Included  (n = 7932) | Excluded  (n = 534) | t/χ^2^ | P |
| --- | --- | --- | --- | --- |
| Age, years old | 30.10 ± 3.90 | 29.89 ± 4.32 | 1.20^a^ | 0.23 |
| GWG | 15.29 ± 4.55 | 15.08 ± 4.57 | 1.01^a^ | 0.31 |
| Ethnicity |  |  | 0.47^b^ | 0.49 |
| Han | 7874 (99.27) | 532 (99.63) |  |  |
| Minorities | 58 (0.73) | 2 (0.37) |  |  |
| Educational level, years^c^ | | | 1.06^b^ | 0.59 |
| ≤9 | 572 (7.23) | 36 (6.76) |  |  |
| 10-12 | 633 (8.00) | 49 (9.19) |  |  |
| >12 | 6705 (84.77) | 448 (84.05) |  |  |
| Occupation |  |  | 4.26^b^ | 0.24 |
| Farmer | 231 (2.91) | 12 (2.25) |  |  |
| Worker | 1838 (23.17) | 136 (25.47) |  |  |
| Others | 4937 (62.24) | 336 (62.92) |  |  |
| None | 926 (11.68) | 50 (9.36) |  |  |
| Gestational hypertensive disorders | | | 0.16^b^ | 0.69 |
| Yes | 479 (6.04) | 30 (5.62) |  |  |
| No | 7453 (93.96) | 504 (94.38) |  |  |
| Gestational diabetes |  |  |  |  |
| Yes | 543 (6.85) | 0 (0.00) | 39.06^b^ | <0.001 |
| No | 7389 (93.15) | 534 (100.00) |  |  |
| GDP |  |  | 1.59^b^ | 0.21 |
| Low | 3965 (49.99) | 282 (52.81) |  |  |
| High | 3967 (50.01) | 252 (47.19) |  |  |
| Population density |  |  | 0.01^b^ | 0.93 |
| Low | 3966 (50.00) | 268 (50.19) |  |  |
| High | 3966 (50.00) | 266 (49.81) |  |  |

Note: Gestational age and season of blood examination were not compared, because only the included pregnant women, who conducted blood examination before delivery, were of gestational age and season at blood examination. ^a^ indicated Student’s t test, ^b^ indicated Chi-square analysis, ^c^ n=7910 for the included pregnant women and n=533 for the excluded pregnant women.


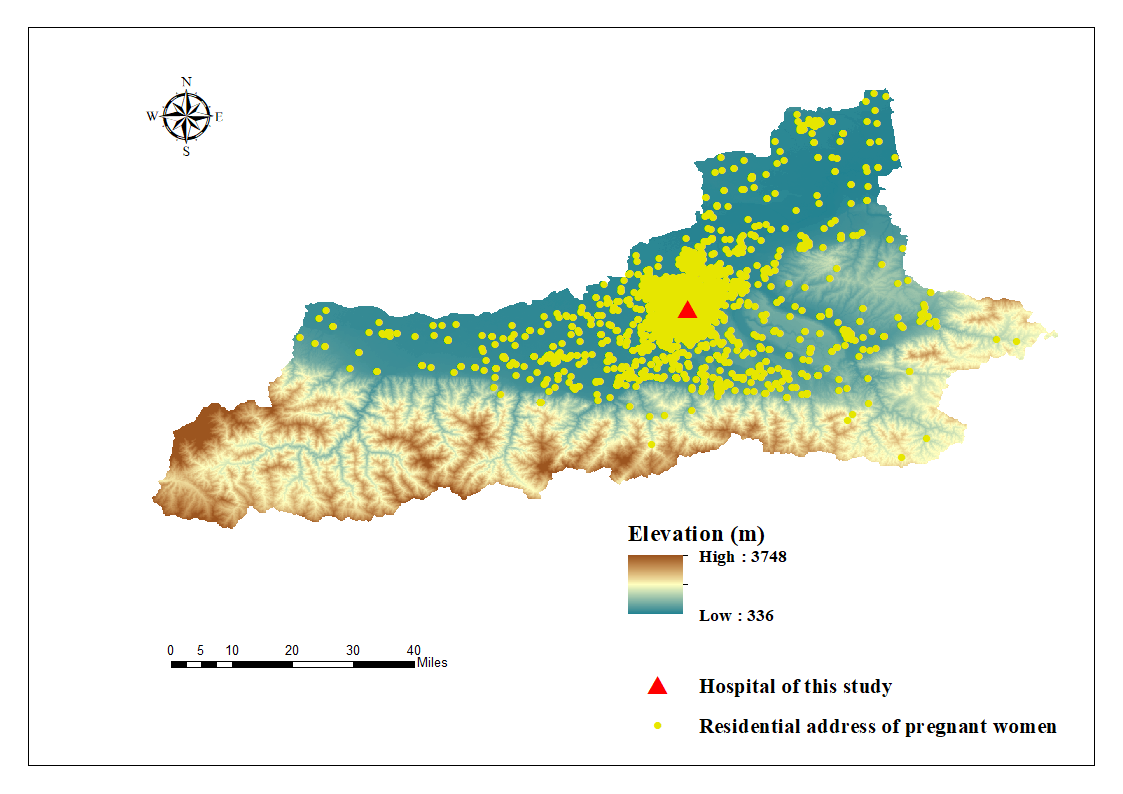


**Fig. S2** Residential address of pregnant women and hospital in the birth cohort


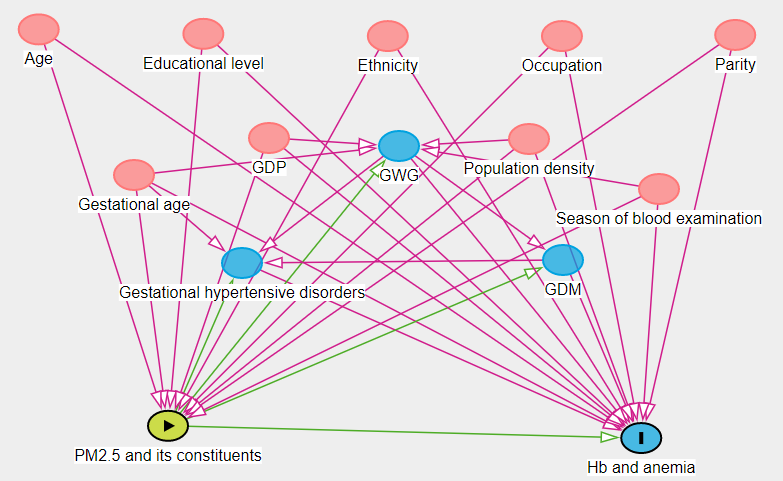


**Fig. S3** Directed acyclic graph for PM_2.5_ and its constituents, Hb, anemia and covariables

**Table S2** Spearman correlations of PM_2.5_ and its constituents (μg/m^3^) of the birth cohort

| Variables | PM_2.5_ | BC | NH_4_^+^ | NO_3_^-^ | OM | SO_4_^2-^ | Dust |
| --- | --- | --- | --- | --- | --- | --- | --- |
| Total |  |  |  |  |  |  |  |
| PM_2.5_ | 1.00 |  |  |  |  |  |  |
| BC | 0.99^a^ | 1.00 |  |  |  |  |  |
| NH_4_^+^ | 0.98^a^ | 0.96^a^ | 1.00 |  |  |  |  |
| NO_3_^-^ | 0.99^a^ | 0.99^a^ | 0.96^a^ | 1.00 |  |  |  |
| OM | 0.97^a^ | 0.98^a^ | 0.94^a^ | 0.97^a^ | 1.00 |  |  |
| SO_4_^2-^ | 0.34^a^ | 0.28^a^ | 0.48^a^ | 0.27^a^ | 0.27^a^ | 1.00 |  |
| Dust | 0.38^a^ | 0.32^a^ | 0.31^a^ | 0.34^a^ | 0.27^a^ | -0.02 | 1.00 |
| Primapara |  |  |  |  |  |  |  |
| PM_2.5_ | 1.00 |  |  |  |  |  |  |
| BC | 0.99^a^ | 1.00 |  |  |  |  |  |
| NH_4_^+^ | 0.98^a^ | 0.96^a^ | 1.00 |  |  |  |  |
| NO_3_^-^ | 0.99^a^ | 0.99^a^ | 0.96^a^ | 1.00 |  |  |  |
| OM | 0.97^a^ | 0.98^a^ | 0.94^a^ | 0.97^a^ | 1.00 |  |  |
| SO_4_^2-^ | 0.31^a^ | 0.25^a^ | 0.46^a^ | 0.23^a^ | 0.24^a^ | 1.00 |  |
| Dust | 0.37^a^ | 0.31^a^ | 0.30^a^ | 0.33^a^ | 0.25^a^ | -0.03^a^ | 1.00 |
| Multipara |  |  |  |  |  |  |  |
| PM_2.5_ | 1.00 |  |  |  |  |  |  |
| BC | 0.99^a^ | 1.00 |  |  |  |  |  |
| NH_4_^+^ | 0.98^a^ | 0.97^a^ | 1.00 |  |  |  |  |
| NO_3_^-^ | 0.99^a^ | 0.99^a^ | 0.97^a^ | 1.00 |  |  |  |
| OM | 0.98^a^ | 0.98^a^ | 0.95^a^ | 0.98^a^ | 1.00 |  |  |
| SO_4_^2-^ | 0.39^a^ | 0.34^a^ | 0.51^a^ | 0.33^a^ | 0.33^a^ | 1.00 |  |
| Dust | 0.40^a^ | 0.34^a^ | 0.33^a^ | 0.36^a^ | 0.30^a^ | -0.01 | 1.00 |

Note: ^a^ indicated P < 0.05.

**Table S3** Stratified analysis by sociodemographic variables on the association between PM_2.5_ (per IQR increase, μg/m^3^) and Hb

| Subgroups | Primapara | |  | Multipara | |
| --- | --- | --- | --- | --- | --- |
|  | β (95% CI) | P for interaction |  | β (95% CI) | P for interaction |
| Age, years old |  |  |  |  |  |
| 20-24.9 | 0.73 (-0.85, 2.31) | Reference |  | 0.64 (-5.42, 6.69) | Reference |
| 25-29.9 | 0.42 (-0.20, 1.05) | 0.88 |  | 0.74 (-1.11, 2.59) | 0.59 |
| 30-34.9 | -0.53 (-1.32, 0.27) | 0.19 |  | -1.15 (-2.26, -0.05) | 0.29 |
| ≥ 35 | 0.01 (-1.88, 1.90) | 0.77 |  | -1.21 (-2.45, 0.03) | 0.25 |
| Gestational age, weeks |  |  |  |  |  |
| < 37 | -0.86 (-2.11, 0.40) | 0.06 |  | -0.05 (-1.97, 1.87) | 0.23 |
| ≥ 37 | 0.26 (-0.22, 0.75) | Reference |  | -0.94 (-1.76, -0.12) | Reference |
| GWG, kg |  |  |  |  |  |
| < 12.5 | 0.23 (-0.72, 1.18) | Reference |  | -0.29 (-1.58, 1.01) | Reference |
| 12.5-15 | -0.02 (-0.81, 0.77) | 0.77 |  | -0.67 (-1.87, 0.52) | 0.34 |
| >15 | 0.18 (-0.51, 0.86) | 0.93 |  | -1.37 (-2.77, 0.03) | 0.49 |
| Ethnicity |  |  |  |  |  |
| Han | 0.12 (-0.33, 0.58) | Reference |  | -0.80 (-1.54, -0.05) | Reference |
| Minorities | -3.31 (-9.75, 3.12) | 0.30 |  | - ^a^ | 0.03 |
| Educational level, years |  |  |  |  |  |
| ≤9 | 0.75 (-1.40, 2.90) | Reference |  | 1.06 (-1.30, 3.43) | Reference |
| 10-12 | 1.13 (-0.50, 2.75) | 0.74 |  | -0.90 (-3.32, 1.52) | 0.32 |
| >12 | -0.02 (-0.50, 0.47) | 0.60 |  | -1.01 (-1.84, -0.18) | 0.26 |
| Occupation |  |  |  |  |  |
| Farmer | 1.85 (-2.11, 5.81) | Reference |  | - ^a^ | Reference |
| Worker | -0.47 (-1.41, 0.46) | 0.32 |  | -0.65 (-2.47, 1.17) | 0.24 |
| Others | 0.29 (-0.29, 0.86) | 0.57 |  | -0.58 (-1.51, 0.36) | 0.14 |
| None | 0.07 (-1.18, 1.33) | 0.46 |  | -1.07 (-3.02, 0.88) | 0.24 |
| Gestational hypertensive disorders | |  |  |  |  |
| Yes | -1.33 (-3.44, 0.79) | 0.10 |  | -0.45 (-4.14, 3.23) | 0.995 |
| No | 0.21 (-0.26, 0.67) | Reference |  | -0.74 (-1.49, 0.02) | Reference |
| Gestational diabetes |  |  |  |  |  |
| Yes | -0.73 (-2.43, 0.96) | 0.15 |  | - ^a^ | 0.38 |
| No | 0.17 (-0.30, 0.64) | Reference |  | -0.86 (-1.64, -0.08) | Reference |
| GDP |  |  |  |  |  |
| Low | 0.11 (-0.53, 0.75) | Reference |  | -0.35 (-1.44, 0.74) | Reference |
| High | 0.10 (-0.54, 0.74) | 0.96 |  | -1.12 (-2.15, -0.10) | 0.41 |
| Population density |  |  |  |  |  |
| Low | -0.11 (-0.74, 0.53) | Reference |  | -0.25 (-1.34, 0.84) | Reference |
| High | 0.35 (-0.30, 1.00) | 0.44 |  | -1.21 (-2.24, -0.19) | 0.32 |
| Season of blood examination | |  |  |  |  |
| Warm season | 0.17 (-0.40, 0.73) | Reference |  | -0.24 (-1.21, 0.72) | Reference |
| Cold season | -0.02 (-0.79, 0.75) | 0.71 |  | -1.60 (-2.78, -0.43) | 0.048 |

Note: Adjusted for age, gestational age, GWG, ethnicity, educational level, occupation, gestational hypertensive disorders, gestational diabetes, GDP, population density and season of blood examination, without each category variable. ^a^ model was cannot be fitted because the sample size was less than or equal to the number of model parameters.

**Table S4** Stratified analysis by sociodemographic variables on the association between BC (per IQR increase, μg/m^3^) and Hb

| Subgroups | Primapara | |  | Multipara | |
| --- | --- | --- | --- | --- | --- |
|  | β (95% CI) | P for interaction |  | β (95% CI) | P for interaction |
| Age, years old |  |  |  |  |  |
| 20-24.9 | 0.95 (-0.90, 2.81) | Reference |  | 0.52 (-6.91, 7.96) | Reference |
| 25-29.9 | 0.41 (-0.31, 1.13) | 0.78 |  | 0.71 (-1.30, 2.71) | 0.56 |
| 30-34.9 | -0.59 (-1.51, 0.33) | 0.18 |  | -1.20 (-2.39, -0.01) | 0.30 |
| ≥ 35 | 0.20 (-2.02, 2.41) | 0.84 |  | -1.44 (-2.79, -0.09) | 0.24 |
| Gestational age, weeks |  |  |  |  |  |
| < 37 | -0.90 (-2.31, 0.51) | 0.09 |  | -0.004 (-2.03, 2.02) | 0.19 |
| ≥ 37 | 0.27 (-0.30, 0.83) | Reference |  | -1.07 (-1.96, -0.18) | Reference |
| GWG, kg |  |  |  |  |  |
| < 12.5 | 0.44 (-0.67, 1.55) | Reference |  | -0.53 (-1.95, 0.88) | Reference |
| 12.5-15 | -0.14 (-1.04, 0.76) | 0.52 |  | -0.84 (-2.15, 0.47) | 0.36 |
| >15 | 0.15 (-0.64, 0.94) | 0.82 |  | -1.17 (-2.68, 0.34) | 0.87 |
| Ethnicity |  |  |  |  |  |
| Han | 0.12 (-0.41, 0.64) | Reference |  | -0.89 (-1.70, -0.08) | Reference |
| Minorities | -3.15 (-10.56, 4.26) | 0.41 |  | - ^a^ | 0.04 |
| Educational level, years |  |  |  |  |  |
| ≤9 | 0.89 (-1.66, 3.43) | Reference |  | 0.97 (-1.58, 3.52) | Reference |
| 10-12 | 1.60 (-0.29, 3.48) | 0.60 |  | -0.74 (-3.41, 1.94) | 0.44 |
| >12 | -0.07 (-0.63, 0.49) | 0.59 |  | -1.13 (-2.03, -0.24) | 0.32 |
| Occupation |  |  |  |  |  |
| Farmer | 2.75 (-1.76, 7.27) | Reference |  | - ^a^ | Reference |
| Worker | -0.52 (-1.59, 0.55) | 0.25 |  | -0.70 (-2.67, 1.26) | 0.27 |
| Others | 0.28 (-0.39, 0.95) | 0.46 |  | -0.68 (-1.70, 0.34) | 0.18 |
| None | 0.02 (-1.44, 1.47) | 0.36 |  | -1.12 (-3.21, 0.97) | 0.26 |
| Gestational hypertensive disorders | |  |  |  |  |
| Yes | -1.22 (-3.62, 1.19) | 0.21 |  | -0.43 (-4.31, 3.46) | 0.94 |
| No | 0.20 (-0.34, 0.73) | Reference |  | -0.83 (-1.65, -0.01) | Reference |
| Gestational diabetes |  |  |  |  |  |
| Yes | -0.64 (-2.61, 1.33) | 0.24 |  | - ^a^ | 0.44 |
| No | 0.16 (-0.39, 0.70) | Reference |  | -0.95 (-1.80, -0.10) | Reference |
| GDP |  |  |  |  |  |
| Low | 0.15 (-0.59, 0.89) | Reference |  | -0.48 (-1.66, 0.71) | Reference |
| High | 0.06 (-0.68, 0.80) | 0.94 |  | -1.18 (-2.29, -0.07) | 0.52 |
| Population density |  |  |  |  |  |
| Low | -0.11 (-0.84, 0.63) | Reference |  | -0.41 (-1.60, 0.77) | Reference |
| High | 0.34 (-0.41, 1.08) | 0.57 |  | -1.25 (-2.36, -0.14) | 0.46 |
| Season of blood examination | |  |  |  |  |
| Warm season | 0.12 (-0.57, 0.81) | Reference |  | -0.34 (-1.46, 0.77) | Reference |
| Cold season | 0.07 (-0.74, 0.88) | 0.92 |  | -1.46 (-2.64, -0.29) | 0.11 |

Note: Adjusted for age, gestational age, GWG, ethnicity, educational level, occupation, gestational hypertensive disorders, gestational diabetes, GDP, population density and season of blood examination, without each category variable. ^a^ model was cannot be fitted because the sample size was less than or equal to the number of model parameters.

**Table S5** Stratified analysis by sociodemographic variables on the association between NH_4_^+^ (per IQR increase, μg/m^3^) and Hb

| Subgroups | Primapara | |  | Multipara | |
| --- | --- | --- | --- | --- | --- |
|  | β (95% CI) | P for interaction |  | β (95% CI) | P for interaction |
| Age, years old |  |  |  |  |  |
| 20-24.9 | 0.66 (-0.74, 2.07) | Reference |  | 0.41 (-4.76, 5.58) | Reference |
| 25-29.9 | 0.40 (-0.15, 0.95) | 0.80 |  | 0.59 (-1.05, 2.23) | 0.45 |
| 30-34.9 | -0.51 (-1.21, 0.19) | 0.13 |  | -1.02 (-1.99, -0.05) | 0.17 |
| ≥ 35 | 0.05 (-1.61, 1.70) | 0.71 |  | -0.98 (-2.07, 0.10) | 0.17 |
| Gestational age, weeks |  |  |  |  |  |
| < 37 | -0.87 (-2.02, 0.28) | 0.04 |  | -0.17 (-1.92, 1.57) | 0.34 |
| ≥ 37 | 0.24 (-0.19, 0.67) | Reference |  | -0.78 (-1.49, -0.07) | Reference |
| GWG, kg |  |  |  |  |  |
| < 12.5 | 0.19 (-0.66, 1.04) | Reference |  | -0.32 (-1.47, 0.84) | Reference |
| 12.5-15 | -0.08 (-0.77, 0.62) | 0.67 |  | -0.51 (-1.57, 0.54) | 0.63 |
| >15 | 0.21 (-0.39, 0.82) | 0.88 |  | -1.22 (-2.44, 0.002) | 0.46 |
| Ethnicity |  |  |  |  |  |
| Han | 0.11 (-0.29, 0.51) | Reference |  | -0.70 (-1.35, -0.04) | Reference |
| Minorities | -3.29 (-8.66, 2.08) | 0.25 |  | - ^a^ | 0.03 |
| Educational level, years |  |  |  |  |  |
| ≤9 | 0.73 (-1.16, 2.63) | Reference |  | 1.04 (-1.07, 3.14) | Reference |
| 10-12 | 1.01 (-0.44, 2.46) | 0.78 |  | -0.61 (-2.76, 1.54) | 0.22 |
| >12 | -0.02 (-0.44, 0.41) | 0.51 |  | -0.92 (-1.65, -0.20) | 0.10 |
| Occupation |  |  |  |  |  |
| Farmer | 1.65 (-1.87, 5.16) | Reference |  | - ^a^ | Reference |
| Worker | -0.41 (-1.23, 0.42) | 0.24 |  | -0.58 (-2.18, 1.02) | 0.22 |
| Others | 0.26 (-0.25, 0.76) | 0.44 |  | -0.48 (-1.30, 0.34) | 0.14 |
| None | 0.03 (-1.09, 1.16) | 0.36 |  | -1.04 (-2.75, 0.68) | 0.27 |
| Gestational hypertensive disorders | |  |  |  |  |
| Yes | -1.04 (-2.96, 0.87) | 0.13 |  | -0.87 (-4.16, 2.43) | 0.83 |
| No | 0.17 (-0.24, 0.58) | Reference |  | -0.62 (-1.28, 0.05) | Reference |
| Gestational diabetes |  |  |  |  |  |
| Yes | -0.62 (-2.12, 0.88) | 0.18 |  | - ^a^ | 0.40 |
| No | 0.16 (-0.26, 0.57) | Reference |  | -0.75 (-1.44, -0.06) | Reference |
| GDP |  |  |  |  |  |
| Low | 0.13 (-0.45, 0.70) | Reference |  | -0.33 (-1.30, 0.64) | Reference |
| High | 0.08 (-0.48, 0.64) | 0.95 |  | -0.94 (-1.83, -0.04) | 0.44 |
| Population density |  |  |  |  |  |
| Low | -0.04 (-0.60, 0.53) | Reference |  | -0.24 (-1.21, 0.73) | Reference |
| High | 0.25 (-0.31, 0.82) | 0.53 |  | -1.03 (-1.92, -0.13) | 0.29 |
| Season of blood examination | |  |  |  |  |
| Warm season | 0.15 (-0.36, 0.66) | Reference |  | -0.18 (-1.06, 0.70) | Reference |
| Cold season | 0.01 (-0.64, 0.65) | 0.73 |  | -1.32 (-2.30, -0.33) | 0.06 |

Note: Adjusted for age, gestational age, GWG, ethnicity, educational level, occupation, gestational hypertensive disorders, gestational diabetes, GDP, population density and season of blood examination, without each category variable. ^a^ model was cannot be fitted because the sample size was less than or equal to the number of model parameters.

**Table S6** Stratified analysis by sociodemographic variables on the association between NO_3_^-^ (per IQR increase, μg/m^3^) and Hb

| Subgroups | Primapara | |  | Multipara | |
| --- | --- | --- | --- | --- | --- |
|  | β (95% CI) | P for interaction |  | β (95% CI) | P for interaction |
| Age, years old |  |  |  |  |  |
| 20-24.9 | 0.96 (-0.84, 2.76) | Reference |  | 2.31 (-5.31, 9.94) | Reference |
| 25-29.9 | 0.45 (-0.24, 1.13) | 0.78 |  | 0.89 (-0.99, 2.77) | 0.46 |
| 30-34.9 | -0.59 (-1.46, 0.28) | 0.16 |  | -1.26 (-2.40, -0.13) | 0.19 |
| ≥ 35 | -0.02 (-2.11, 2.06) | 0.68 |  | -1.33 (-2.61, -0.05) | 0.16 |
| Gestational age, weeks |  |  |  |  |  |
| < 37 | -1.00 (-2.37, 0.36) | 0.05 |  | -0.10 (-2.07, 1.87) | 0.24 |
| ≥ 37 | 0.28 (-0.26, 0.81) | Reference |  | -0.97 (-1.81, -0.13) | Reference |
| GWG, kg |  |  |  |  |  |
| < 12.5 | 0.38 (-0.68, 1.44) | Reference |  | -0.34 (-1.68, 1.00) | Reference |
| 12.5-15 | -0.06 (-0.92, 0.79) | 0.61 |  | -0.78 (-2.00, 0.46) | 0.32 |
| >15 | 0.12 (-0.64, 0.87) | 0.83 |  | -1.30 (-2.74, 0.14) | 0.60 |
| Ethnicity |  |  |  |  |  |
| Han | 0.11 (-0.39, 0.61) | Reference |  | -0.84 (-1.61, -0.07) | Reference |
| Minorities | -2.66 (-9.74, 4.43) | 0.42 |  | - ^a^ | 0.03 |
| Educational level, years |  |  |  |  |  |
| ≤9 | 0.79 (-1.72, 3.30) | Reference |  | 1.08 (-1.35, 3.52) | Reference |
| 10-12 | 1.33 (-0.49, 3.14) | 0.68 |  | -0.53 (-3.06, 2.00) | 0.41 |
| >12 | -0.04 (-0.56, 0.49) | 0.64 |  | -1.12 (-1.98, -0.27) | 0.21 |
| Occupation |  |  |  |  |  |
| Farmer | 2.79 (-1.68, 7.25) | Reference |  | - ^a^ | Reference |
| Worker | -0.48 (-1.49, 0.54) | 0.22 |  | -0.70 (-2.56, 1.17) | 0.27 |
| Others | 0.26 (-0.37, 0.90) | 0.39 |  | -0.66 (-1.62, 0.30) | 0.18 |
| None | 0.02 (-1.39, 1.43) | 0.32 |  | -0.98 (-3.01, 1.06) | 0.25 |
| Gestational hypertensive disorders | |  |  |  |  |
| Yes | -1.43 (-3.74, 0.89) | 0.14 |  | -0.28 (-4.03, 3.48) | 0.85 |
| No | 0.20 (-0.31, 0.71) | Reference |  | -0.79 (-1.57, -0.02) | Reference |
| Gestational diabetes |  |  |  |  |  |
| Yes | -0.75 (-2.64, 1.15) | 0.19 |  | - ^a^ | 0.48 |
| No | 0.16 (-0.35, 0.68) | Reference |  | -0.88 (-1.69, -0.08) | Reference |
| GDP |  |  |  |  |  |
| Low | 0.15 (-0.56, 0.87) | Reference |  | -0.45 (-1.60, 0.69) | Reference |
| High | 0.05 (-0.64, 0.74) | 0.89 |  | -1.09 (-2.13, -0.05) | 0.53 |
| Population density |  |  |  |  |  |
| Low | -0.09 (-0.80, 0.62) | Reference |  | -0.42 (-1.56, 0.72) | Reference |
| High | 0.30 (-0.40, 1.00) | 0.58 |  | -1.13 (-2.17, -0.08) | 0.52 |
| Season of blood examination | |  |  |  |  |
| Warm season | 0.13 (-0.51, 0.76) | Reference |  | -0.31 (-1.33, 0.70) | Reference |
| Cold season | 0.05 (-0.76, 0.86) | 0.87 |  | -1.52 (-2.69, -0.34) | 0.08 |

Note: Adjusted for age, gestational age, GWG, ethnicity, educational level, occupation, gestational hypertensive disorders, gestational diabetes, GDP, population density and season of blood examination, without each category variable. ^a^ model was cannot be fitted because the sample size was less than or equal to the number of model parameters.

**Table S7** Stratified analysis by sociodemographic variables on the association between OM (per IQR increase, μg/m^3^) and Hb

| Subgroups | Primapara | |  | Multipara | |
| --- | --- | --- | --- | --- | --- |
|  | β (95% CI) | P for interaction |  | β (95% CI) | P for interaction |
| Age, years old |  |  |  |  |  |
| 20-24.9 | 0.64 (-0.93, 2.21) | Reference |  | -0.01 (-6.45, 6.43) | Reference |
| 25-29.9 | 0.27 (-0.37, 0.90) | 0.94 |  | 0.47 (-1.27, 2.21) | 0.73 |
| 30-34.9 | -0.78 (-1.59, 0.02) | 0.19 |  | -1.16 (-2.19, -0.12) | 0.46 |
| ≥ 35 | 0.13 (-1.78, 2.04) | 0.996 |  | -1.00 (-2.18, 0.17) | 0.42 |
| Gestational age, weeks |  |  |  |  |  |
| < 37 | -0.91 (-2.12, 0.31) | 0.10 |  | 0.14 (-1.60, 1.89) | 0.13 |
| ≥ 37 | 0.09 (-0.40, 0.58) | Reference |  | -0.97 (-1.75, -0.20) | Reference |
| GWG, kg |  |  |  |  |  |
| < 12.5 | 0.19 (-0.79, 1.16) | Reference |  | -0.47 (-1.70, 0.75) | Reference |
| 12.5-15 | -0.25 (-1.04, 0.53) | 0.62 |  | -0.58 (-1.72, 0.56) | 0.40 |
| >15 | 0.002 (-0.69, 0.69) | 0.93 |  | -1.21 (-2.51, 0.10) | 0.75 |
| Ethnicity |  |  |  |  |  |
| Han | -0.04 (-0.50, 0.42) | Reference |  | -0.79 (-1.49, -0.09) | Reference |
| Minorities | -3.68 (-10.01, 2.66) | 0.37 |  | - ^a^ | 0.004 |
| Educational level, years |  |  |  |  |  |
| ≤9 | 0.63 (-1.54, 2.79) | Reference |  | 1.33 (-0.89, 3.55) | Reference |
| 10-12 | 1.20 (-0.41, 2.81) | 0.60 |  | -0.68 (-3.00, 1.64) | 0.28 |
| >12 | -0.21 (-0.69, 0.28) | 0.57 |  | -1.06 (-1.84, -0.28) | 0.16 |
| Occupation |  |  |  |  |  |
| Farmer | 2.06 (-1.81, 5.92) | Reference |  | - ^a^ | Reference |
| Worker | -0.58 (-1.52, 0.35) | 0.40 |  | -0.52 (-2.24, 1.20) | 0.48 |
| Others | 0.11 (-0.48, 0.69) | 0.67 |  | -0.63 (-1.52, 0.25) | 0.37 |
| None | -0.08 (-1.35, 1.18) | 0.55 |  | -1.19 (-3.00, 0.63) | 0.53 |
| Gestational hypertensive disorders | |  |  |  |  |
| Yes | -1.16 (-3.23, 0.91) | 0.23 |  | -0.57 (-3.89, 2.76) | 0.94 |
| No | 0.02 (-0.44, 0.49) | Reference |  | -0.72 (-1.44, -0.01) | Reference |
| Gestational diabetes |  |  |  |  |  |
| Yes | -0.61 (-2.30, 1.09) | 0.29 |  | - ^a^ | 0.50 |
| No | -0.01 (-0.49, 0.46) | Reference |  | -0.81 (-1.55, -0.08) | Reference |
| GDP |  |  |  |  |  |
| Low | -0.04 (-0.67, 0.60) | Reference |  | -0.37 (-1.39, 0.65) | Reference |
| High | -0.07 (-0.73, 0.58) | 0.97 |  | -1.07 (-2.05, -0.09) | 0.46 |
| Population density |  |  |  |  |  |
| Low | -0.24 (-0.87, 0.40) | Reference |  | -0.31 (-1.33, 0.72) | Reference |
| High | 0.15 (-0.51, 0.81) | 0.59 |  | -1.15 (-2.12, -0.18) | 0.40 |
| Season of blood examination | |  |  |  |  |
| Warm season | -0.11 (-0.72, 0.51) | Reference |  | -0.24 (-1.22, 0.74) | Reference |
| Cold season | -0.01 (-0.69, 0.67) | 0.84 |  | -1.32 (-2.31, -0.32) | 0.10 |

Note: Adjusted for age, gestational age, GWG, ethnicity, educational level, occupation, gestational hypertensive disorders, gestational diabetes, GDP, population density and season of blood examination, without each category variable. ^a^ model was cannot be fitted because the sample size was less than or equal to the number of model parameters.

**Table S8** Stratified analysis by sociodemographic variables on the association between SO_4_^2-^ (per IQR increase, μg/m^3^) and Hb

| Subgroups | Primapara | |  | Multipara | |
| --- | --- | --- | --- | --- | --- |
|  | β (95% CI) | P for interaction |  | β (95% CI) | P for interaction |
| Age, years old |  |  |  |  |  |
| 20-24.9 | 0.15 (-1.51, 1.82) | Reference |  | -1.11 (-4.53, 2.31) | Reference |
| 25-29.9 | 0.42 (-0.25, 1.09) | 0.88 |  | -0.53 (-2.55, 1.49) | 0.55 |
| 30-34.9 | -0.55 (-1.49, 0.39) | 0.30 |  | -0.44 (-1.60, 0.72) | 0.35 |
| ≥ 35 | 0.32 (-1.76, 2.40) | 0.997 |  | -0.09 (-1.39, 1.22) | 0.57 |
| Gestational age, weeks |  |  |  |  |  |
| < 37 | -0.64 (-2.20, 0.91) | 0.23 |  | -0.48 (-2.56, 1.60) | 0.90 |
| ≥ 37 | 0.26 (-0.27, 0.79) | Reference |  | -0.38 (-1.23, 0.47) | Reference |
| GWG, kg |  |  |  |  |  |
| < 12.5 | -0.38 (-1.46, 0.71) | Reference |  | -0.34 (-1.77, 1.09) | Reference |
| 12.5-15 | -0.17 (-1.04, 0.71) | 0.93 |  | 0.34 (-0.94, 1.61) | 0.09 |
| >15 | 0.65 (-0.09, 1.39) | 0.20 |  | -1.31 (-2.68, 0.05) | 0.32 |
| Ethnicity |  |  |  |  |  |
| Han | 0.18 (-0.32, 0.68) | Reference |  | -0.41 (-1.18, 0.37) | Reference |
| Minorities | -6.11 (-11.67, -0.55) | 0.10 |  | - ^a^ | 0.19 |
| Educational level, years |  |  |  |  |  |
| ≤9 | 0.90 (-1.08, 2.88) | Reference |  | 0.91 (-1.25, 3.08) | Reference |
| 10-12 | 0.54 (-1.24, 2.31) | 0.78 |  | -0.95 (-3.21, 1.31) | 0.06 |
| >12 | 0.05 (-0.50, 0.59) | 0.38 |  | -0.56 (-1.46, 0.35) | 0.03 |
| Occupation |  |  |  |  |  |
| Farmer | -0.55 (-3.95, 2.84) | Reference |  | - ^a^ | Reference |
| Worker | -0.36 (-1.40, 0.68) | 0.69 |  | -0.29 (-2.17, 1.58) | 0.45 |
| Others | 0.39 (-0.25, 1.03) | 0.97 |  | 0.03 (-0.97, 1.04) | 0.45 |
| None | 0.11 (-1.26, 1.48) | 0.90 |  | -1.56 (-3.45, 0.33) | 0.99 |
| Gestational hypertensive disorders | |  |  |  |  |
| Yes | 0.01 (-2.34, 2.37) | 0.49 |  | -3.36 (-7.19, 0.47) | 0.14 |
| No | 0.17 (-0.34, 0.68) | Reference |  | -0.17 (-0.96, 0.62) | Reference |
| Gestational diabetes |  |  |  |  |  |
| Yes | -0.57 (-2.42, 1.27) | 0.46 |  | - ^a^ | 0.36 |
| No | 0.22 (-0.30, 0.74) | Reference |  | -0.51 (-1.33, 0.30) | Reference |
| GDP |  |  |  |  |  |
| Low | 0.09 (-0.59, 0.77) | Reference |  | 0.03 (-1.04, 1.10) | Reference |
| High | 0.25 (-0.49, 1.00) | 0.80 |  | -0.81 (-1.97, 0.35) | 0.28 |
| Population density |  |  |  |  |  |
| Low | 0.12 (-0.55, 0.79) | Reference |  | 0.31 (-0.75, 1.38) | Reference |
| High | 0.23 (-0.52, 0.98) | 0.56 |  | -1.18 (-2.33, -0.03) | 0.03 |
| Season of blood examination | |  |  |  |  |
| Warm season | 0.37 (-0.34, 1.08) | Reference |  | 0.37 (-0.79, 1.54) | Reference |
| Cold season | -0.10 (-0.81, 0.61) | 0.38 |  | -1.02 (-2.06, 0.02) | 0.06 |

Note: Adjusted for age, gestational age, GWG, ethnicity, educational level, occupation, gestational hypertensive disorders, gestational diabetes, GDP, population density and season of blood examination, without each category variable. ^a^ model was cannot be fitted because the sample size was less than or equal to the number of model parameters.

**Table S9** Stratified analysis by sociodemographic variables on the association between Dust (per IQR increase, μg/m^3^) and Hb

| Subgroups | Primapara | |  | Multipara | |
| --- | --- | --- | --- | --- | --- |
|  | β (95% CI) | P for interaction |  | β (95% CI) | P for interaction |
| Age, years old |  |  |  |  |  |
| 20-24.9 | 0.20 (-1.50, 1.91) | Reference |  | 1.41 (-3.50, 6.31) | Reference |
| 25-29.9 | 0.49 (-0.16, 1.14) | 0.79 |  | 1.22 (-0.65, 3.10) | 0.80 |
| 30-34.9 | 0.60 (-0.28, 1.49) | 0.75 |  | -0.18 (-1.29, 0.93) | 0.999 |
| ≥ 35 | -0.73 (-2.82, 1.36) | 0.54 |  | -0.92 (-2.20, 0.35) | 0.69 |
| Gestational age, weeks |  |  |  |  |  |
| < 37 | 0.20 (-1.33, 1.73) | 0.41 |  | -0.09 (-2.28, 2.10) | 0.62 |
| ≥ 37 | 0.47 (-0.04, 0.98) | Reference |  | -0.24 (-1.04, 0.57) | Reference |
| GWG, kg |  |  |  |  |  |
| < 12.5 | -0.002 (-1.00, 1.00) | Reference |  | 1.01 (-0.32, 2.34) | Reference |
| 12.5-15 | 0.95 (0.11, 1.79) | 0.14 |  | -0.54 (-1.77, 0.69) | 0.02 |
| >15 | 0.35 (-0.39, 1.09) | 0.43 |  | -0.85 (-2.22, 0.52) | 0.09 |
| Ethnicity |  |  |  |  |  |
| Han | 0.45 (-0.04, 0.93) | Reference |  | -0.14 (-0.90, 0.61) | Reference |
| Minorities | 1.21 (-4.99, 7.42) | 0.47 |  | - ^a^ | 0.41 |
| Educational level, years |  |  |  |  |  |
| ≤9 | 0.28 (-1.92, 2.47) | Reference |  | -0.51 (-2.74, 1.72) | Reference |
| 10-12 | -0.27 (-2.11, 1.57) | 0.68 |  | -1.96 (-4.22, 0.30) | 0.97 |
| >12 | 0.50 (-0.02, 1.02) | 0.71 |  | 0.26 (-0.60, 1.12) | 0.06 |
| Occupation |  |  |  |  |  |
| Farmer | -1.10 (-4.82, 2.62) | Reference |  | - ^a^ | Reference |
| Worker | 0.01 (-0.99, 1.02) | 0.71 |  | -0.44 (-2.28, 1.41) | 0.13 |
| Others | 0.59 (-0.03, 1.21) | 0.46 |  | 0.02 (-0.93, 0.97) | 0.03 |
| None | 0.64 (-0.67, 1.95) | 0.57 |  | 0.42 (-1.52, 2.37) | 0.04 |
| Gestational hypertensive disorders | |  |  |  |  |
| Yes | -1.86 (-4.13, 0.41) | 0.01 |  | 2.31 (-1.61, 6.22) | 0.41 |
| No | 0.60 (0.11, 1.09) | Reference |  | -0.26 (-1.02, 0.51) | Reference |
| Gestational diabetes |  |  |  |  |  |
| Yes | -1.09 (-2.98, 0.79) | 0.04 |  | - ^a^ | 0.27 |
| No | 0.55 (0.04, 1.05) | Reference |  | -0.32 (-1.12, 0.48) | Reference |
| GDP |  |  |  |  |  |
| Low | 0.23 (-0.43, 0.89) | Reference |  | 0.24 (-0.81, 1.30) | Reference |
| High | 0.66 (-0.05, 1.38) | 0.43 |  | -0.69 (-1.78, 0.40) | 0.32 |
| Population density |  |  |  |  |  |
| Low | -0.04 (-0.70, 0.62) | Reference |  | 0.45 (-0.61, 1.52) | Reference |
| High | 1.01 (0.30, 1.72) | 0.08 |  | -0.81 (-1.88, 0.26) | 0.29 |
| Season of blood examination | |  |  |  |  |
| Warm season | 0.71 (0.15, 1.28) | Reference |  | -0.20 (-1.11, 0.71) | Reference |
| Cold season | -0.39 (-1.35, 0.56) | 0.06 |  | -0.09 (-1.50, 1.33) | 0.78 |

Note: Adjusted for age, gestational age, GWG, ethnicity, educational level, occupation, gestational hypertensive disorders, gestational diabetes, GDP, population density and season of blood examination, without each category variable. ^a^ model was cannot be fitted because the sample size was less than or equal to the number of model parameters.

**Table S10** Stratified analysis by sociodemographic variables on the association between PM_2.5_ (per IQR increase, μg/m^3^) and anemia

| Subgroups | Primapara | |  | Multipara | |
| --- | --- | --- | --- | --- | --- |
|  | OR (95% CI) | P for interaction |  | OR (95% CI) | P for interaction |
| Age, years old |  |  |  |  |  |
| 20-24.9 | 0.97 (0.92, 1.02) | Reference |  | 0.87 (0.68, 1.12) | Reference |
| 25-29.9 | 0.99 (0.97, 1.01) | 0.65 |  | 1.01 (0.95, 1.07) | 0.51 |
| 30-34.9 | 1.01 (0.98, 1.03) | 0.17 |  | 1.02 (0.99, 1.06) | 0.47 |
| ≥ 35 | 1.01 (0.95, 1.07) | 0.47 |  | 1.02 (0.98, 1.06) | 0.42 |
| Gestational age, weeks |  |  |  |  |  |
| < 37 | 0.99 (0.95, 1.04) | 0.98 |  | 0.99 (0.93, 1.05) | 0.26 |
| ≥ 37 | 0.99 (0.98, 1.01) | Reference |  | 1.02 (1.00, 1.05) | Reference |
| GWG, kg |  |  |  |  |  |
| < 12.5 | 0.97 (0.94, 1.00) | Reference |  | 0.98 (0.94, 1.03) | Reference |
| 12.5-15 | 1.00 (0.98, 1.03) | 0.14 |  | 1.02 (0.98, 1.06) | 0.17 |
| >15 | 1.00 (0.98, 1.02) | 0.28 |  | 1.05 (1.01, 1.10) | 0.04 |
| Ethnicity |  |  |  |  |  |
| Han | 0.99 (0.98, 1.01) | Reference |  | 1.02 (0.99, 1.04) | Reference |
| Minorities | 0.92 (0.73, 1.16) | 0.71 |  | - ^a^ | 0.12 |
| Educational level, years |  |  |  |  |  |
| ≤9 | 0.97 (0.90, 1.04) | Reference |  | 1.02 (0.95, 1.10) | Reference |
| 10-12 | 0.96 (0.91, 1.02) | 0.99 |  | 1.01 (0.93, 1.09) | 0.71 |
| >12 | 1.00 (0.98, 1.01) | 0.36 |  | 1.02 (0.99, 1.05) | 0.52 |
| Occupation |  |  |  |  |  |
| Farmer | 0.96 (0.85, 1.09) | Reference |  | - ^a^ | Reference |
| Worker | 1.01 (0.98, 1.04) | 0.70 |  | 1.00 (0.94, 1.06) | 0.04 |
| Others | 0.99 (0.97, 1.01) | 0.95 |  | 1.01 (0.98, 1.04) | 0.04 |
| None | 0.99 (0.95, 1.03) | 0.99 |  | 1.02 (0.95, 1.09) | 0.06 |
| Gestational hypertensive disorders | |  |  |  |  |
| Yes | 1.00 (0.94, 1.06) | 0.92 |  | 1.01 (0.92, 1.11) | 0.95 |
| No | 0.99 (0.98, 1.01) | Reference |  | 1.02 (0.99, 1.04) | Reference |
| Gestational diabetes |  |  |  |  |  |
| Yes | 1.03 (0.98, 1.08) | 0.04 |  | - ^a^ | 0.02 |
| No | 0.99 (0.98, 1.01) | Reference |  | 1.02 (1.00, 1.05) | Reference |
| GDP |  |  |  |  |  |
| Low | 0.99 (0.97, 1.01) | Reference |  | 1.01 (0.98, 1.05) | Reference |
| High | 1.00 (0.98, 1.02) | 0.63 |  | 1.02 (0.98, 1.05) | 0.88 |
| Population density |  |  |  |  |  |
| Low | 0.99 (0.97, 1.02) | Reference |  | 1.00 (0.97, 1.04) | Reference |
| High | 0.99 (0.97, 1.01) | 0.95 |  | 1.03 (1.00, 1.06) | 0.38 |
| Season of blood examination | |  |  |  |  |
| Warm season | 0.99 (0.98, 1.01) | Reference |  | 1.01 (0.98, 1.04) | Reference |
| Cold season | 1.00 (0.97, 1.02) | 0.96 |  | 1.03 (0.99, 1.07) | 0.25 |

Note: Adjusted for age, gestational age, GWG, ethnicity, educational level, occupation, gestational hypertensive disorders, gestational diabetes, GDP, population density and season of blood examination, without each category variable. ^a^ model was cannot be fitted because the sample size was less than or equal to the number of model parameters.

**Table S11** Stratified analysis by sociodemographic variables on the association between BC (per IQR increase, μg/m^3^) and anemia

| Subgroups | Primapara | |  | Multipara | |
| --- | --- | --- | --- | --- | --- |
|  | OR (95% CI) | P for interaction |  | OR (95% CI) | P for interaction |
| Age, years old |  |  |  |  |  |
| 20-24.9 | 0.96 (0.91, 1.02) | Reference |  | 0.86 (0.63, 1.17) | Reference |
| 25-29.9 | 0.99 (0.96, 1.01) | 0.60 |  | 1.01 (0.95, 1.09) | 0.59 |
| 30-34.9 | 1.01 (0.98, 1.04) | 0.15 |  | 1.02 (0.98, 1.06) | 0.61 |
| ≥ 35 | 1.01 (0.94, 1.08) | 0.52 |  | 1.02 (0.98, 1.07) | 0.54 |
| Gestational age, weeks |  |  |  |  |  |
| < 37 | 0.99 (0.94, 1.04) | 0.88 |  | 0.99 (0.93, 1.05) | 0.21 |
| ≥ 37 | 0.99 (0.98, 1.01) | Reference |  | 1.03 (1.00, 1.06) | Reference |
| GWG, kg |  |  |  |  |  |
| < 12.5 | 0.96 (0.93, 0.99) | Reference |  | 0.99 (0.94, 1.04) | Reference |
| 12.5-15 | 1.01 (0.98, 1.04) | 0.08 |  | 1.02 (0.97, 1.06) | 0.22 |
| >15 | 1.00 (0.97, 1.02) | 0.23 |  | 1.05 (1.00, 1.10) | 0.12 |
| Ethnicity |  |  |  |  |  |
| Han | 0.99 (0.98, 1.01) | Reference |  | 1.02 (0.99, 1.05) | Reference |
| Minorities | 0.88 (0.68, 1.14) | 0.88 |  | - ^a^ | 0.11 |
| Educational level, years |  |  |  |  |  |
| ≤9 | 0.96 (0.88, 1.04) | Reference |  | 1.03 (0.95, 1.12) | Reference |
| 10-12 | 0.95 (0.89, 1.02) | 0.97 |  | 1.00 (0.92, 1.09) | 0.59 |
| >12 | 1.00 (0.98, 1.02) | 0.30 |  | 1.02 (0.99, 1.05) | 0.42 |
| Occupation |  |  |  |  |  |
| Farmer | 0.94 (0.81, 1.08) | Reference |  | - ^a^ | Reference |
| Worker | 1.01 (0.98, 1.05) | 0.61 |  | 0.99 (0.93, 1.06) | 0.04 |
| Others | 0.99 (0.97, 1.01) | 0.93 |  | 1.01 (0.98, 1.05) | 0.04 |
| None | 0.99 (0.94, 1.04) | 0.83 |  | 1.02 (0.95, 1.09) | 0.05 |
| Gestational hypertensive disorders | |  |  |  |  |
| Yes | 1.00 (0.93, 1.07) | 0.77 |  | 1.00 (0.91, 1.11) | 0.91 |
| No | 0.99 (0.98, 1.01) | Reference |  | 1.02 (0.99, 1.05) | Reference |
| Gestational diabetes |  |  |  |  |  |
| Yes | 1.03 (0.98, 1.09) | 0.06 |  | - ^a^ | 0.01 |
| No | 0.99 (0.97, 1.01) | Reference |  | 1.03 (1.00, 1.06) | Reference |
| GDP |  |  |  |  |  |
| Low | 0.99 (0.96, 1.01) | Reference |  | 1.02 (0.98, 1.06) | Reference |
| High | 1.00 (0.98, 1.02) | 0.64 |  | 1.02 (0.98, 1.05) | 0.88 |
| Population density |  |  |  |  |  |
| Low | 0.99 (0.97, 1.02) | Reference |  | 1.01 (0.97, 1.05) | Reference |
| High | 0.99 (0.97, 1.02) | 0.87 |  | 1.03 (0.99, 1.06) | 0.63 |
| Season of blood examination | |  |  |  |  |
| Warm season | 0.99 (0.97, 1.02) | Reference |  | 1.01 (0.97, 1.05) | Reference |
| Cold season | 0.99 (0.97, 1.02) | 0.89 |  | 1.03 (0.99, 1.07) | 0.40 |

Note: Adjusted for age, gestational age, GWG, ethnicity, educational level, occupation, gestational hypertensive disorders, gestational diabetes, GDP, population density and season of blood examination, without each category variable. ^a^ model was cannot be fitted because the sample size was less than or equal to the number of model parameters.

**Table S12** Stratified analysis by sociodemographic variables on the association between NH_4_^+^ (per IQR increase, μg/m^3^) and anemia

| Subgroups | Primapara | |  | Multipara | |
| --- | --- | --- | --- | --- | --- |
|  | OR (95% CI) | P for interaction |  | OR (95% CI) | P for interaction |
| Age, years old |  |  |  |  |  |
| 20-24.9 | 0.97 (0.93, 1.02) | Reference |  | 0.89 (0.72, 1.11) | Reference |
| 25-29.9 | 0.99 (0.97, 1.01) | 0.45 |  | 1.01 (0.95, 1.07) | 0.39 |
| 30-34.9 | 1.01 (0.99, 1.03) | 0.10 |  | 1.02 (0.99, 1.05) | 0.33 |
| ≥ 35 | 1.01 (0.95, 1.06) | 0.37 |  | 1.02 (0.98, 1.05) | 0.31 |
| Gestational age, weeks |  |  |  |  |  |
| < 37 | 1.00 (0.96, 1.04) | 0.78 |  | 0.99 (0.94, 1.05) | 0.33 |
| ≥ 37 | 0.99 (0.98, 1.01) | Reference |  | 1.02 (0.99, 1.04) | Reference |
| GWG, kg |  |  |  |  |  |
| < 12.5 | 0.97 (0.95, 1.00) | Reference |  | 0.99 (0.95, 1.02) | Reference |
| 12.5-15 | 1.01 (0.98, 1.03) | 0.09 |  | 1.01 (0.98, 1.05) | 0.25 |
| >15 | 1.00 (0.98, 1.02) | 0.23 |  | 1.05 (1.01, 1.09) | 0.04 |
| Ethnicity |  |  |  |  |  |
| Han | 1.00 (0.98, 1.01) | Reference |  | 1.02 (0.99, 1.04) | Reference |
| Minorities | 0.93 (0.77, 1.13) | 0.77 |  | - ^a^ | 0.15 |
| Educational level, years |  |  |  |  |  |
| ≤9 | 0.97 (0.91, 1.02) | Reference |  | 1.02 (0.95, 1.09) | Reference |
| 10-12 | 0.97 (0.92, 1.02) | 0.97 |  | 1.00 (0.93, 1.07) | 0.75 |
| >12 | 1.00 (0.99, 1.01) | 0.26 |  | 1.02 (0.99, 1.04) | 0.78 |
| Occupation |  |  |  |  |  |
| Farmer | 0.97 (0.86, 1.08) | Reference |  | - ^a^ | Reference |
| Worker | 1.01 (0.99, 1.04) | 0.53 |  | 1.00 (0.95, 1.05) | 0.04 |
| Others | 0.99 (0.98, 1.01) | 0.83 |  | 1.01 (0.98, 1.04) | 0.04 |
| None | 0.99 (0.95, 1.03) | 0.82 |  | 1.02 (0.96, 1.08) | 0.07 |
| Gestational hypertensive disorders | |  |  |  |  |
| Yes | 1.00 (0.95, 1.06) | 0.99 |  | 1.03 (0.95, 1.12) | 0.63 |
| No | 1.00 (0.98, 1.01) | Reference |  | 1.01 (0.99, 1.04) | Reference |
| Gestational diabetes |  |  |  |  |  |
| Yes | 1.03 (0.99, 1.07) | 0.06 |  | - ^a^ | 0.03 |
| No | 0.99 (0.98, 1.01) | Reference |  | 1.02 (1.00, 1.05) | Reference |
| GDP |  |  |  |  |  |
| Low | 0.99 (0.97, 1.01) | Reference |  | 1.01 (0.98, 1.05) | Reference |
| High | 1.00 (0.98, 1.02) | 0.44 |  | 1.01 (0.99. 1.05) | 0.93 |
| Population density |  |  |  |  |  |
| Low | 0.99 (0.98, 1.01) | Reference |  | 1.00 (0.99, 1.03) | Reference |
| High | 1.00 (0.98, 1.01) | 0.73 |  | 1.03 (1.00, 1.06) | 0.32 |
| Season of blood examination | |  |  |  |  |
| Warm season | 1.00 (0.98, 1.01) | Reference |  | 1.00 (0.98, 1.03) | Reference |
| Cold season | 1.00 (0.98, 1.02) | 0.99 |  | 1.03 (0.99, 1.06) | 0.24 |

Note: Adjusted for age, gestational age, GWG, ethnicity, educational level, occupation, gestational hypertensive disorders, gestational diabetes, GDP, population density and season of blood examination, without each category variable. ^a^ model was cannot be fitted because the sample size was less than or equal to the number of model parameters.

**Table S13** Stratified analysis by sociodemographic variables on the association between NO_3_^-^ (per IQR increase, μg/m^3^) and anemia

| Subgroups | Primapara | |  | Multipara | |
| --- | --- | --- | --- | --- | --- |
|  | OR (95% CI) | P for interaction |  | OR (95% CI) | P for interaction |
| Age, years old |  |  |  |  |  |
| 20-24.9 | 0.97 (0.91, 1.03) | Reference |  | 0.80 (0.59, 1.10) | Reference |
| 25-29.9 | 0.98 (0.96, 1.01) | 0.68 |  | 1.00 (0.94, 1.07) | 0.51 |
| 30-34.9 | 1.01 (0.98, 1.04) | 0.15 |  | 1.02 (0.99, 1.06) | 0.44 |
| ≥ 35 | 1.01 (0.95, 1.08) | 0.43 |  | 1.02 (0.98, 1.07) | 0.39 |
| Gestational age, weeks |  |  |  |  |  |
| < 37 | 1.00 (0.95, 1.04) | 0.93 |  | 1.00 (0.94, 1.06) | 0.33 |
| ≥ 37 | 0.99 (0.98, 1.01) | Reference |  | 1.02 (0.99, 1.05) | Reference |
| GWG, kg |  |  |  |  |  |
| < 12.5 | 0.96 (0.93, 1.00) | Reference |  | 0.98 (0.94, 1.03) | Reference |
| 12.5-15 | 1.01 (0.98, 1.03) | 0.10 |  | 1.02 (0.98, 1.06) | 0.16 |
| >15 | 1.00 (0.97, 1.02) | 0.22 |  | 1.05 (1.01, 1.10) | 0.06 |
| Ethnicity |  |  |  |  |  |
| Han | 0.99 (0.98, 1.01) | Reference |  | 1.02 (0.99, 1.04) | Reference |
| Minorities | 0.88 (0.69, 1.13) | 0.85 |  | - ^a^ | 0.12 |
| Educational level, years |  |  |  |  |  |
| ≤9 | 0.96 (0.89, 1.04) | Reference |  | 1.02 (0.94, 1.11) | Reference |
| 10-12 | 0.96 (0.90, 1.03) | 0.86 |  | 1.00 (0.92, 1.08) | 0.63 |
| >12 | 1.00 (0.98, 1.02) | 0.32 |  | 1.02 (0.99, 1.05) | 0.59 |
| Occupation |  |  |  |  |  |
| Farmer | 0.92 (0.80, 1.07) | Reference |  | - ^a^ | Reference |
| Worker | 1.01 (0.98, 1.05) | 0.44 |  | 1.00 (0.94, 1.06) | 0.04 |
| Others | 0.99 (0.97, 1.01) | 0.70 |  | 1.01 (0.98, 1.05) | 0.04 |
| None | 0.99 (0.94, 1.04) | 0.66 |  | 1.01 (0.94, 1.09) | 0.047 |
| Gestational hypertensive disorders | |  |  |  |  |
| Yes | 1.00 (0.94, 1.07) | 0.89 |  | 1.00 (0.91, 1.10) | 0.84 |
| No | 0.99 (0.98, 1.01) | Reference |  | 1.02 (0.99, 1.04) | Reference |
| Gestational diabetes |  |  |  |  |  |
| Yes | 1.04 (0.98, 1.09) | 0.048 |  | - ^a^ | 0.02 |
| No | 0.99 (0.97, 1.01) | Reference |  | 1.03 (1.00, 1.05) | Reference |
| GDP |  |  |  |  |  |
| Low | 0.99 (0.97, 1.01) | Reference |  | 1.02 (0.98, 1.06) | Reference |
| High | 1.00 (0.98, 1.02) | 0.60 |  | 1.02 (0.98, 1.05) | 0.95 |
| Population density |  |  |  |  |  |
| Low | 0.99 (0.97, 1.02) | Reference |  | 1.01 (0.97, 1.05) | Reference |
| High | 0.99 (0.97, 1.01) | 0.93 |  | 1.03 (0.99, 1.06) | 0.56 |
| Season of blood examination | |  |  |  |  |
| Warm season | 0.99 (0.97, 1.01) | Reference |  | 1.01 (0.98, 1.04) | Reference |
| Cold season | 0.99 (0.97, 1.02) | 0.96 |  | 1.03 (0.99, 1.07) | 0.29 |

Note: Adjusted for age, gestational age, GWG, ethnicity, educational level, occupation, gestational hypertensive disorders, gestational diabetes, GDP, population density and season of blood examination, without each category variable. ^a^ model was cannot be fitted because the sample size was less than or equal to the number of model parameters.

**Table S14** Stratified analysis by sociodemographic variables on the association between OM (per IQR increase, μg/m^3^) and anemia

| Subgroups | Primapara | |  | Multipara | |
| --- | --- | --- | --- | --- | --- |
|  | OR (95% CI) | P for interaction |  | OR (95% CI) | P for interaction |
| Age, years old |  |  |  |  |  |
| 20-24.9 | 0.98 (0.93, 1.03) | Reference |  | 0.88 (0.68, 1.15) | Reference |
| 25-29.9 | 0.99 (0.97, 1.01) | 0.69 |  | 1.02 (0.96, 1.08) | 0.73 |
| 30-34.9 | 1.02 (0.99, 1.04) | 0.12 |  | 1.02 (0.99, 1.06) | 0.81 |
| ≥ 35 | 1.00 (0.94, 1.06) | 0.74 |  | 1.02 (0.98, 1.06) | 0.75 |
| Gestational age, weeks |  |  |  |  |  |
| < 37 | 1.00 (0.96, 1.04) | 0.91 |  | 0.99 (0.94, 1.05) | 0.22 |
| ≥ 37 | 1.00 (0.98, 1.02) | Reference |  | 1.02 (1.00, 1.05) | Reference |
| GWG, kg |  |  |  |  |  |
| < 12.5 | 0.97 (0.94, 1.00) | Reference |  | 0.99 (0.95, 1.03) | Reference |
| 12.5-15 | 1.02 (0.99, 1.04) | 0.06 |  | 1.02 (0.98, 1.06) | 0.22 |
| >15 | 1.00 (0.98, 1.03) | 0.21 |  | 1.05 (1.01, 1.09) | 0.09 |
| Ethnicity |  |  |  |  |  |
| Han | 1.00 (0.99, 1.01) | Reference |  | 1.02 (0.99, 1.04) | Reference |
| Minorities | 0.94 (0.74, 1.18) | 0.72 |  | - ^a^ | 0.03 |
| Educational level, years |  |  |  |  |  |
| ≤9 | 0.96 (0.90, 1.03) | Reference |  | 1.02 (0.95, 1.09) | Reference |
| 10-12 | 0.97 (0.92, 1.03) | 0.70 |  | 1.00 (0.93, 1.08) | 0.69 |
| >12 | 1.00 (0.99, 1.02) | 0.17 |  | 1.02 (0.99, 1.05) | 0.60 |
| Occupation |  |  |  |  |  |
| Farmer | 0.95 (0.84, 1.08) | Reference |  | - ^a^ | Reference |
| Worker | 1.02 (0.99, 1.05) | 0.82 |  | 0.99 (0.94, 1.05) | 0.07 |
| Others | 0.99 (0.98, 1.01) | 0.78 |  | 1.01 (0.98, 1.04) | 0.08 |
| None | 1.00 (0.95, 1.04) | 0.88 |  | 1.03 (0.96, 1.09) | 0.14 |
| Gestational hypertensive disorders | |  |  |  |  |
| Yes | 1.00 (0.95, 1.06) | 0.76 |  | 1.01 (0.93, 1.10) | 0.98 |
| No | 1.00 (0.98, 1.02) | Reference |  | 1.02 (0.99, 1.04) | Reference |
| Gestational diabetes |  |  |  |  |  |
| Yes | 1.03 (0.99, 1.08) | 0.06 |  | - ^a^ | 0.02 |
| No | 1.00 (0.98, 1.01) | Reference |  | 1.02 (1.00, 1.05) | Reference |
| GDP |  |  |  |  |  |
| Low | 1.00 (0.98, 1.02) | Reference |  | 1.02 (0.98, 1.05) | Reference |
| High | 1.00 (0.98, 1.03) | 0.77 |  | 1.02 (0.98, 1.05) | 0.999 |
| Population density |  |  |  |  |  |
| Low | 1.00 (0.98, 1.02) | Reference |  | 1.01 (0.97, 1.04) | Reference |
| High | 1.00 (0.98, 1.02) | 0.84 |  | 1.03 (0.99, 1.06) | 0.55 |
| Season of blood examination | |  |  |  |  |
| Warm season | 1.00 (0.98, 1.02) | Reference |  | 1.01 (0.98, 1.04) | Reference |
| Cold season | 1.00 (0.98, 1.02) | 0.83 |  | 1.03 (0.99, 1.06) | 0.40 |

Note: Adjusted for age, gestational age, GWG, ethnicity, educational level, occupation, gestational hypertensive disorders, gestational diabetes, GDP, population density and season of blood examination, without each category variable. ^a^ model was cannot be fitted because the sample size was less than or equal to the number of model parameters.

**Table S15** Stratified analysis by sociodemographic variables on the association between SO_4_^2-^ (per IQR increase, μg/m^3^) and anemia

| Subgroups | Primapara | |  | Multipara | |
| --- | --- | --- | --- | --- | --- |
|  | OR (95% CI) | P for interaction |  | OR (95% CI) | P for interaction |
| Age, years old |  |  |  |  |  |
| 20-24.9 | 0.96 (0.91, 1.02) | Reference |  | 0.98 (0.85, 1.14) | Reference |
| 25-29.9 | 1.00 (0.98, 1.02) | 0.11 |  | 1.04 (0.97, 1.11) | 0.29 |
| 30-34.9 | 1.01 (0.98, 1.04) | 0.13 |  | 1.00 (0.97, 1.04) | 0.27 |
| ≥ 35 | 0.99 (0.92, 1.05) | 0.46 |  | 1.00 (0.96, 1.04) | 0.38 |
| Gestational age, weeks |  |  |  |  |  |
| < 37 | 1.01 (0.96, 1.07) | 0.38 |  | 0.99 (0.93, 1.06) | 0.61 |
| ≥ 37 | 1.00 (0.98, 1.01) | Reference |  | 1.01 (0.98, 1.04) | Reference |
| GWG, kg |  |  |  |  |  |
| < 12.5 | 0.99 (0.96, 1.03) | Reference |  | 0.98 (0.93, 1.03) | Reference |
| 12.5-15 | 1.01 (0.98, 1.04) | 0.34 |  | 1.00 (0.95, 1.04) | 0.85 |
| >15 | 0.99 (0.97, 1.02) | 0.63 |  | 1.04 (1.00, 1.09) | 0.09 |
| Ethnicity |  |  |  |  |  |
| Han | 1.00 (0.98, 1.01) | Reference |  | 1.01 (0.98, 1.03) | Reference |
| Minorities | 1.04 (0.84, 1.29) | 0.60 |  | - ^a^ | 0.64 |
| Educational level, years |  |  |  |  |  |
| ≤9 | 0.96 (0.90, 1.03) | Reference |  | 1.00 (0.93, 1.08) | Reference |
| 10-12 | 0.96 (0.90, 1.02) | 0.71 |  | 1.00 (0.93, 1.07) | 0.69 |
| >12 | 1.01 (0.99, 1.02) | 0.31 |  | 1.01 (0.98, 1.04) | 0.37 |
| Occupation |  |  |  |  |  |
| Farmer | 1.06 (0.95, 1.18) | Reference |  | - ^a^ | Reference |
| Worker | 1.02 (0.98, 1.05) | 0.996 |  | 0.99 (0.94, 1.06) | 0.67 |
| Others | 0.99 (0.97, 1.01) | 0.76 |  | 1.00 (0.97, 1.03) | 0.72 |
| None | 0.99 (0.94, 1.03) | 0.63 |  | 1.04 (0.98, 1.11) | 0.85 |
| Gestational hypertensive disorders | |  |  |  |  |
| Yes | 1.00 (0.94, 1.07) | 0.59 |  | 1.16 (1.05, 1.27) | 0.01 |
| No | 1.00 (0.98, 1.01) | Reference |  | 1.00 (0.97, 1.02) | Reference |
| Gestational diabetes |  |  |  |  |  |
| Yes | 1.02 (0.96, 1.07) | 0.66 |  | - ^a^ | 0.40 |
| No | 1.00 (0.98, 1.01) | Reference |  | 1.01 (0.99, 1.04) | Reference |
| GDP |  |  |  |  |  |
| Low | 0.99 (0.97, 1.01) | Reference |  | 1.00 (0.96, 1.03) | Reference |
| High | 1.01 (0.98, 1.03) | 0.24 |  | 1.02 (0.98, 1.06) | 0.51 |
| Population density |  |  |  |  |  |
| Low | 0.99 (0.97, 1.01) | Reference |  | 0.98 (0.95, 1.02) | Reference |
| High | 1.01 (0.99, 1.04) | 0.30 |  | 1.04 (1.00, 1.08) | 0.03 |
| Season of blood examination | |  |  |  |  |
| Warm season | 1.00 (0.97, 1.02) | Reference |  | 0.99 (0.95, 1.03) | Reference |
| Cold season | 1.00 (0.98, 1.02) | 0.80 |  | 1.02 (0.99, 1.06) | 0.22 |

Note: Adjusted for age, gestational age, GWG, ethnicity, educational level, occupation, gestational hypertensive disorders, gestational diabetes, GDP, population density and season of blood examination, without each category variable. ^a^ model was cannot be fitted because the sample size was less than or equal to the number of model parameters.

**Table S16** Stratified analysis by sociodemographic variables on the association between Dust (per IQR increase, μg/m^3^) and anemia

| Subgroups | Primapara | |  | Multipara | |
| --- | --- | --- | --- | --- | --- |
|  | OR (95% CI) | P for interaction |  | OR (95% CI) | P for interaction |
| Age, years old |  |  |  |  |  |
| 20-24.9 | 1.00 (0.95, 1.06) | Reference |  | 0.88 (0.72, 1.08) | Reference |
| 25-29.9 | 0.98 (0.96, 1.00) | 0.47 |  | 0.96 (0.90, 1.03) | 0.66 |
| 30-34.9 | 0.96 (0.94, 0.99) | 0.25 |  | 1.01 (0.97, 1.05) | 0.40 |
| ≥ 35 | 1.05 (0.98, 1.12) | 0.51 |  | 1.02 (0.97, 1.06) | 0.31 |
| Gestational age, weeks |  |  |  |  |  |
| < 37 | 0.95 (0.91, 1.00) | 0.31 |  | 0.98 (0.92, 1.05) | 0.54 |
| ≥ 37 | 0.99 (0.97, 1.00) | Reference |  | 1.00 (0.98, 1.03) | Reference |
| GWG, kg |  |  |  |  |  |
| < 12.5 | 1.00 (0.97, 1.03) | Reference |  | 0.97 (0.92, 1.01) | Reference |
| 12.5-15 | 0.96 (0.94, 0.99) | 0.09 |  | 1.01 (0.97, 1.05) | 0.04 |
| >15 | 0.99 (0.97, 1.01) | 0.53 |  | 1.03 (0.98, 1.07) | 0.05 |
| Ethnicity |  |  |  |  |  |
| Han | 0.98 (0.97, 1.00) | Reference |  | 1.00 (0.98, 1.03) | Reference |
| Minorities | 1.03 (0.83, 1.28) | 0.35 |  | - ^a^ | 0.65 |
| Educational level, years |  |  |  |  |  |
| ≤9 | 1.03 (0.96, 1.11) | Reference |  | 1.01 (0.93, 1.08) | Reference |
| 10-12 | 0.97 (0.91, 1.04) | 0.28 |  | 1.06 (0.99, 1.14) | 0.83 |
| >12 | 0.98 (0.96, 1.00) | 0.12 |  | 0.99 (0.96, 1.02) | 0.11 |
| Occupation |  |  |  |  |  |
| Farmer | 1.05 (0.93, 1.18) | Reference |  | - ^a^ | Reference |
| Worker | 1.00 (0.96, 1.03) | 0.51 |  | 1.03 (0.97, 1.10) | 0.35 |
| Others | 0.98 (0.96, 1.00) | 0.31 |  | 0.99 (0.96, 1.02) | 0.07 |
| None | 0.96 (0.92, 1.00) | 0.26 |  | 0.99 (0.93, 1.06) | 0.11 |
| Gestational hypertensive disorders | |  |  |  |  |
| Yes | 1.00 (0.94, 1.06) | 0.70 |  | 0.93 (0.84, 1.03) | 0.40 |
| No | 0.98 (0.97, 1.00) | Reference |  | 1.00 (0.98, 1.03) | Reference |
| Gestational diabetes |  |  |  |  |  |
| Yes | 1.02 (0.97, 1.07) | 0.06 |  | - ^a^ | 0.19 |
| No | 0.98 (0.96, 1.00) | Reference |  | 1.01 (0.98, 1.03) | Reference |
| GDP |  |  |  |  |  |
| Low | 0.99 (0.96, 1.01) | Reference |  | 0.99 (0.95, 1.02) | Reference |
| High | 0.98 (0.96, 1.00) | 0.59 |  | 1.02 (0.98, 1.06) | 0.21 |
| Population density |  |  |  |  |  |
| Low | 1.00 (0.97, 1.02) | Reference |  | 0.98 (0.94, 1.01) | Reference |
| High | 0.97 (0.95, 0.99) | 0.12 |  | 1.03 (0.99, 1.06) | 0.17 |
| Season of blood examination | |  |  |  |  |
| Warm season | 0.98 (0.96, 1.00) | Reference |  | 1.00 (0.97, 1.03) | Reference |
| Cold season | 1.00 (0.97, 1.03) | 0.28 |  | 1.00 (0.95, 1.05) | 0.81 |

Note: Adjusted for age, gestational age, GWG, ethnicity, educational level, occupation, gestational hypertensive disorders, gestational diabetes, GDP, population density and season of blood examination, without each category variable. ^a^ model was cannot be fitted because the sample size was less than or equal to the number of model parameters.

**Table S17** Distribution of ground monitor based PM_2.5_ (μg/m^3^) of the birth cohort

| Groups | Mean (SD) | IQR | Min | 25th | 50th | 75th | Max |
| --- | --- | --- | --- | --- | --- | --- | --- |
| Primapara | 65.09 (13.26) | 15.86 | 18.05 | 55.86 | 63.61 | 71.72 | 115.62 |
| Multipara | 66.85 (14.94) | 20.08 | 22.33 | 55.62 | 64.70 | 75.70 | 112.72 |

**Table S18** Associations of per IQR increase (μg/m^3^) of ground monitor based PM_2.5_ with Hb and anemia

| Outcomes | Primapara | |  | Multipara | |
| --- | --- | --- | --- | --- | --- |
|  | β/OR (95% CI) | P |  | β/OR (95% CI) | P |
| Hb |  |  |  |  |  |
| Crude model | 0.21 (-0.21, 0.64) | 0.32 |  | -0.91 (-1.63, -0.19) | 0.01 |
| Adjusted model^a^ | 0.25 (-0.18, 0.69) | 0.26 |  | -0.89 (-1.63, -0.14) | 0.02 |
| Anemia |  |  |  |  |  |
| Crude model | 0.96 (0.89, 1.04) | 0.34 |  | 1.11 (0.99, 1.24) | 0.08 |
| Adjusted model^a^ | 0.96 (0.88, 1.04) | 0.30 |  | 1.09 (0.97, 1.23) | 0.16 |

Note: ^a^ Adjusted for age, gestational age, GWG, ethnicity, educational level, occupation, gestational hypertensive disorders, gestational diabetes, GDP, population density and season of blood examination.

**Table S19** Stratified analysis by sociodemographic variables on the association between ground monitor based PM_2.5_ (per IQR increase, μg/m^3^) and Hb

| Subgroups | Primapara | |  | Multipara | |
| --- | --- | --- | --- | --- | --- |
|  | β (95% CI) | P for interaction |  | β (95% CI) | P for interaction |
| Age, years old |  |  |  |  |  |
| 20-24.9 | 1.01 (-0.61, 2.62) | Reference |  | 3.51 (-2.78, 9.80) | Reference |
| 25-29.9 | 0.57 (-0.03, 1.17) | 0.89 |  | 0.78 (-1.03, 2.59) | 0.18 |
| 30-34.9 | -0.44 (-1.22, 0.34) | 0.19 |  | -1.16 (-2.26, -0.06) | 0.06 |
| ≥ 35 | 0.60 (-1.21, 2.41) | 0.94 |  | -1.81 (-3.05, -0.56) | 0.03 |
| Gestational age, weeks |  |  |  |  |  |
| < 37 | -1.00 (-2.19, 0.20) | 0.02 |  | 0.02 (-1.97, 2.02) | 0.21 |
| ≥ 37 | 0.46 (-0.01, 0.93) | Reference |  | -1.08 (-1.89, -0.27) | Reference |
| GWG, kg |  |  |  |  |  |
| < 12.5 | 0.49 (-0.42, 1.41) | Reference |  | -0.87 (-2.19, 0.45) | Reference |
| 12.5-15 | 0.24 (-0.52, 0.99) | 0.77 |  | -0.69 (-1.89, 0.50) | 0.65 |
| >15 | 0.16 (-0.51, 0.83) | 0.66 |  | -1.12 (-2.50, 0.27) | 0.93 |
| Ethnicity |  |  |  |  |  |
| Han | 0.27 (-0.17, 0.71) | Reference |  | -0.93 (-1.67, -0.18) | Reference |
| Minorities | -1.59 (-7.20, 4.01) | 0.45 |  | - ^a^ | 0.06 |
| Educational level, years |  |  |  |  |  |
| ≤9 | 0.88 (-1.37, 3.13) | Reference |  | 1.38 (-0.91, 3.67) | Reference |
| 10-12 | 1.15 (-0.49, 2.78) | 0.78 |  | -0.26 (-2.63, 2.11) | 0.37 |
| >12 | 0.15 (-0.31, 0.62) | 0.63 |  | -1.36 (-2.19, -0.52) | 0.08 |
| Occupation |  |  |  |  |  |
| Farmer | 0.77 (-3.26, 4.80) | Reference |  | - ^a^ | Reference |
| Worker | -0.43 (-1.33, 0.47) | 0.75 |  | -1.17 (-2.97, 0.62) | 0.25 |
| Others | 0.48 (-0.08, 1.03) | 0.84 |  | -0.65 (-1.59, 0.29) | 0.10 |
| None | 0.36 (-0.89, 1.62) | 0.96 |  | -0.86 (-2.79, 1.07) | 0.14 |
| Gestational hypertensive disorders | |  |  |  |  |
| Yes | -1.10 (-3.09, 0.88) | 0.11 |  | -0.80 (-4.42, 2.81) | 0.84 |
| No | 0.36 (-0.09, 0.81) | Reference |  | -0.86 (-1.62, -0.11) | Reference |
| Gestational diabetes |  |  |  |  |  |
| Yes | -0.46 (-2.16, 1.24) | 0.23 |  | - ^a^ | 0.64 |
| No | 0.31 (-0.15, 0.76) | Reference |  | -0.95 (-1.73, -0.17) | Reference |
| GDP |  |  |  |  |  |
| Low | 0.29 (-0.33, 0.91) | Reference |  | -0.55 (-1.63, 0.53) | Reference |
| High | 0.20 (-0.42, 0.83) | 0.90 |  | -1.21 (-2.25, -0.17) | 0.50 |
| Population density |  |  |  |  |  |
| Low | 0.16 (-0.45, 0.77) | Reference |  | -0.46 (-1.53, 0.62) | Reference |
| High | 0.36 (-0.27, 0.99) | 0.86 |  | -1.31 (-2.34, -0.27) | 0.39 |
| Season of blood examination | |  |  |  |  |
| Warm season | 0.25 (-0.30, 0.80) | Reference |  | -0.46 (-1.43, 0.50) | Reference |
| Cold season | 0.26 (-0.46, 0.98) | 0.99 |  | -1.63 (-2.80, -0.45) | 0.10 |

Note: Adjusted for age, gestational age, GWG, ethnicity, educational level, occupation, gestational hypertensive disorders, gestational diabetes, GDP, population density and season of blood examination, without each category variable. ^a^ model was cannot be fitted because the sample size was less than or equal to the number of model parameters.

**Table S20** Stratified analysis by sociodemographic variables on the association between ground monitor based PM_2.5_ (per IQR increase, μg/m^3^) and anemia

| Subgroups | Primapara | |  | Multipara | |
| --- | --- | --- | --- | --- | --- |
|  | OR (95% CI) | P for interaction |  | OR (95% CI) | P for interaction |
| Age, years old |  |  |  |  |  |
| 20-24.9 | 0.97 (0.92, 1.02) | Reference |  | 0.80 (0.62, 1.03) | Reference |
| 25-29.9 | 0.98 (0.96, 1.00) | 0.66 |  | 1.00 (0.94, 1.06) | 0.26 |
| 30-34.9 | 1.01 (0.99, 1.04) | 0.10 |  | 1.02 (0.99, 1.06) | 0.21 |
| ≥ 35 | 1.00 (0.94, 1.06) | 0.55 |  | 1.03 (0.99, 1.08) | 0.14 |
| Gestational age, weeks |  |  |  |  |  |
| < 37 | 1.00 (0.96, 1.05) | 0.51 |  | 1.00 (0.94, 1.07) | 0.46 |
| ≥ 37 | 0.99 (0.98, 1.01) | Reference |  | 1.02 (0.99, 1.05) | Reference |
| GWG, kg |  |  |  |  |  |
| < 12.5 | 0.96 (0.94, 0.99) | Reference |  | 0.99 (0.95, 1.04) | Reference |
| 12.5-15 | 1.00 (0.97, 1.02) | 0.13 |  | 1.01 (0.97, 1.05) | 0.32 |
| >15 | 1.00 (0.98, 1.02) | 0.10 |  | 1.05 (1.00, 1.10) | 0.10 |
| Ethnicity |  |  |  |  |  |
| Han | 0.99 (0.98, 1.01) | Reference |  | 1.02 (0.99, 1.04) | Reference |
| Minorities | 0.88 (0.73, 1.07) | 0.77 |  | -^a^ | 0.25 |
| Educational level, years |  |  |  |  |  |
| ≤9 | 0.95 (0.89, 1.02) | Reference |  | 0.99(0.92, 1.07) | Reference |
| 10-12 | 0.97 (0.92, 1.03) | 0.51 |  | 1.00 (0.92, 1.07) | 0.999 |
| >12 | 1.00 (0.98, 1.01) | 0.17 |  | 1.03 (1.00, 1.05) | 0.80 |
| Occupation |  |  |  |  |  |
| Farmer | 0.94 (0.82, 1.07) | Reference |  | -^a^ | Reference |
| Worker | 1.02 (0.99, 1.05) | 0.40 |  | 1.00 (0.95, 1.06) | 0.049 |
| Others | 0.99 (0.97, 1.00) | 0.74 |  | 1.02 (0.99, 1.05) | 0.04 |
| None | 0.98 (0.94, 1.02) | 0.78 |  | 1.00 (0.93, 1.07) | 0.03 |
| Gestational hypertensive disorders | |  |  |  |  |
| Yes | 0.99 (0.94, 1.05) | 0.81 |  | 1.01 (0.92, 1.10) | 0.99 |
| No | 0.99 (0.98, 1.01) | Reference |  | 1.02 (0.99, 1.04) | Reference |
| Gestational diabetes |  |  |  |  |  |
| Yes | 1.03 (0.98, 1.08) | 0.07 |  | -^a^ | 0.049 |
| No | 0.99 (0.98, 1.00) | Reference |  | 1.02 (1.00, 1.05) | Reference |
| GDP |  |  |  |  |  |
| Low | 0.99 (0.97, 1.01) | Reference |  | 1.02 (0.98, 1.06) | Reference |
| High | 1.00 (0.98, 1.02) | 0.54 |  | 1.02 (0.98, 1.05) | 0.94 |
| Population density |  |  |  |  |  |
| Low | 0.99 (0.97, 1.01) | Reference |  | 1.01 (0.97, 1.04) | Reference |
| High | 0.99 (0.98, 1.01) | 0.66 |  | 1.03 (1.00, 1.07) | 0.43 |
| Season of blood examination | |  |  |  |  |
| Warm season | 0.99 (0.98, 1.01) | Reference |  | 1.00 (0.97, 1.04) | Reference |
| Cold season | 0.99 (0.97, 1.01) | 0.80 |  | 1.04 (1.00, 1.08) | 0.14 |

Note: Adjusted for age, gestational age, GWG, ethnicity, educational level, occupation, gestational hypertensive disorders, gestational diabetes, GDP, population density and season of blood examination, without each category variable. ^a^ model was cannot be fitted because the sample size was less than or equal to the number of model parameters.

**Table S21** Lag effect of ground monitor based PM_2.5_ (per IQR increase, μg/m^3^) on Hb and anemia

| Models | Primapara | |  | Multipara | |
| --- | --- | --- | --- | --- | --- |
|  | β/OR (95% CI) | P |  | β/OR (95% CI) | P |
| Hb |  |  |  |  |  |
| Lag01 | 0.01 (-0.20, 0.21) | 0.95 |  | -0.17 (-0.56, 0.22) | 0.39 |
| Lag02 | 0.03 (-0.19, 0.26) | 0.76 |  | -0.25 (-0.66, 0.16) | 0.23 |
| Lag03 | 0.03 (-0.20, 0.26) | 0.79 |  | -0.35 (-0.75, 0.06) | 0.09 |
| Lag04 | -0.01 (-0.23, 0.21) | 0.92 |  | -0.35 (-0.75, 0.04) | 0.08 |
| Anemia |  |  |  |  |  |
| Lag01 | 0.99 (0.95, 1.03) | 0.65 |  | 1.06 (0.99, 1.12) | 0.08 |
| Lag02 | 0.98 (0.94, 1.03) | 0.47 |  | 1.06 (0.99, 1.13) | 0.10 |
| Lag03 | 0.99 (0.95, 1.04) | 0.78 |  | 1.05 (0.99, 1.12) | 0.12 |
| Lag04 | 1.00 (0.96, 1.05) | 0.89 |  | 1.05 (0.98, 1.11) | 0.17 |

Note: Adjusted for age, gestational age, GWG, ethnicity, educational level, occupation, gestational hypertensive disorders, gestational diabetes, GDP, population density and season of blood examination.


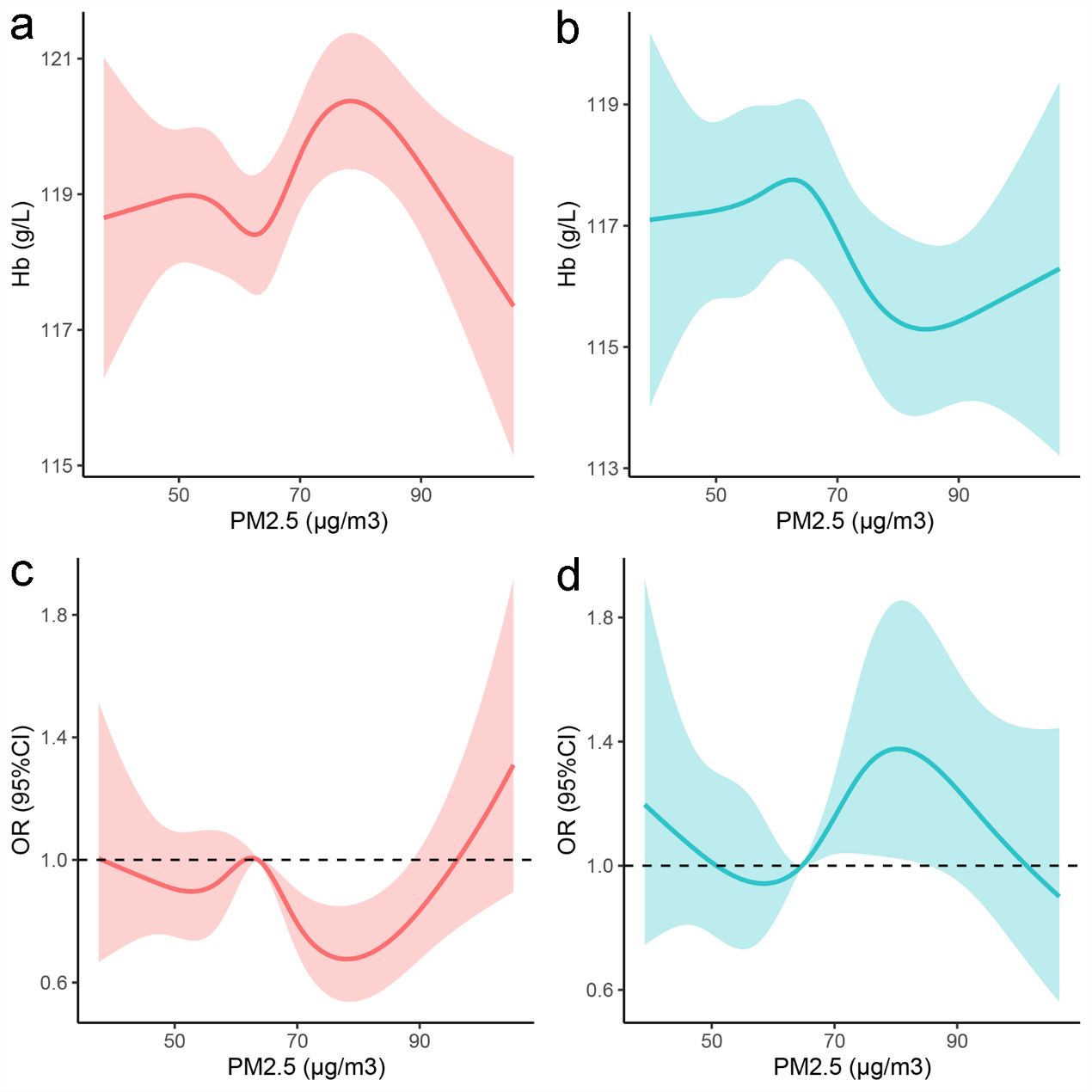


**Fig. S4** Non-linear associations of ground monitor based PM_2.5_ with Hb and anemia. The non-linear associations of ground monitor based PM_2.5_ with Hb in primapara (a) and multipara (b). The non-linear associations of ground monitor based PM_2.5_ with anemia in primapara (c) and multipara (d). Adjusted for age, gestational age, GWG, ethnicity, educational level, occupation, gestational hypertensive disorders, gestational diabetes, GDP, population density and season of blood examination.
